# Fertility History and Physical and Mental Health Changes in European Older Adults

**DOI:** 10.1007/s10680-018-9489-x

**Published:** 2018-04-26

**Authors:** Katherine Keenan, Emily Grundy

**Affiliations:** 1 Department of Geography and Sustainable Development, University of St. Andrews, Irvine Building, North Street, St. Andrews, KY16 9AL UK; 2 Institute for Social and Economic Research, University of Essex, Wivenoe Park, Colchester, CO4 3SQ UK

**Keywords:** Older adults, Fertility history, Health changes, Survey of Health, Ageing and Retirement in Europe

## Abstract

Previous studies have shown that aspects of reproductive history, such as earlier parenthood and high parity, are associated with poorer health in mid and later life. However, it is unclear which dimensions of health are most affected by reproductive history, and whether the pattern of associations varies for measures of physical, psychological and cognitive health. Such variation might provide more insight into possible underlying mechanisms. We use longitudinal data for men and women aged 50–79 years in ten European countries from the Survey of Health, Ageing and Retirement in Europe to analyse associations between completed fertility history and self-reported and observed health indicators measured 2–3 years apart (functional limitations, chronic diseases, grip strength, depression and cognition), adjusting for socio-demographic, and health factors at baseline. Using multiple imputation and pattern mixture modelling, we tested the robustness of estimates to missing data mechanisms. The results are partly consistent with previous studies and show that women who became mothers before age 20 had worse functional health at baseline and were more likely to suffer functional health declines. Parents of 4 or more children had worse physical, psychological and cognitive health at baseline and were more likely to develop circulatory disease over the follow-up period. Men who delayed fatherhood until age 35 or later had better health at baseline but did not experience significantly different health declines. This study improves our understanding of linkages between fertility histories and later life health and possible implications of changes in fertility patterns for population health. However, research ideally using prospective life course data is needed to further elucidate possible mechanisms, considering interactions with partnership histories, health behaviour patterns and socio-economic trajectories.

**Electronic supplementary material:**

The online version of this article (10.1007/s10680-018-9489-x) contains supplementary material, which is available to authorized users.

## Introduction

Studies investigating the life course determinants of health in older age have found that aspects of reproductive history, such as timing of entry to parenthood and completed family size, are associated with post-reproductive morbidity and mortality (Doblhammer 2000; Einiö et al. 2015, 2016; Grundy and Kravdal 2010; Grundy and Tomassini 2005; Hank 2010). Specifically, having no or in some contexts many children (four or five or more) and, among parents, earlier age at first birth, is associated with poorer later life physical (Barban 2013; Grundy and Read 2015; Grundy and Tomassini 2005; Hank 2010; Henretta 2007) and in some studies mental health (Kalil and Kunz 2002; Kravdal et al. 2017; Mirowsky and Ross 2002; Read et al. 2011). The mechanisms that underlie these associations may be complex; involving a combination of social and biological selection processes and biosocial and socio-economic life course influences on health (Grundy and Read 2015), which in some cases may be offsetting. For example, parenthood may motivate positive lifestyle changes and children, as well as providing a source of social control on unhealthy behaviours, are also a potential source of social support which may be especially valuable at older ages; these factors would suggest benefits of having more children. However, the physiological stress of repeated pregnancy and parturition also present challenges for women and both women and men may suffer health damaging consequences from the cumulated stress and financial strain associated with large family size. Generalised biosocial effects of balances between costs and benefits of childbearing and rearing might be expected to influence all indicators of health. However, there may also be pathways specific to certain types of conditions. For example, it is well established that childlessness and delayed motherhood increase women’s risks of breast and reproductive system cancers (Kvåle et al. 1994). It has also been suggested that high parity may be associated with higher risks of musculoskeletal, metabolic and cardiovascular disease among women because the physiological challenges posed by repeated pregnancy, parturition and lactation may affect bone density (Christensen and Vaupel 1996; Pirkle et al. 2014), glucose metabolism and obesity which is a known risk factor for cardiovascular disease (Sowers 2003). Examining a range of outcomes and comparing results for women and men, as we do in this study, may help to distinguish specific from general effects of fertility trajectories on later life health. There is also likely to be a complex dynamic relationship between fertility and partnership history over the life course which exerts differential effects on later life health (Kravdal et al. 2012; Steele et al. 2005).

The overall aim of this study is to investigate associations between fertility history and changes in a range of physical and mental health indicators (both observed and self-reported) measured over a 2–3 year period among European adults aged 50–79 years. This extends previous research in four ways. First, although studies have used a variety of different self-reported indicators of poor health (including functional limitations, disability, self-reported health and mental health) (Barban 2013; Grundy and Tomassini 2005; Henretta et al. 2008) few have compared multiple physical and mental health indicators (Hank 2010; Mirowsky 2005). Second, few have assessed the association using objective measures of morbidity (Grundy and Read 2015; Guralnik et al. 2009; Lawlor et al. 2003; McMunn et al. 2016) and in this study we include an objectively measured indicator of physical health strongly predictive of future disability, grip strength (Rantanen et al. 1999). Third, whereas most previous studies have been cross-sectional (with some exceptions, (Grundy and Read 2015; Read et al. 2011), we conduct a prospective analysis of changes in health, allowing us to assess whether particular fertility patterns precipitate larger declines in health among the over 50s. Fourth, we exploit the cross-national aspect of our data to investigate the hypothesis that the fertility–health nexus might vary contextually (Grundy 2009; Grundy and Foverskov 2016).

## Background

### First Birth Timing and Later Life Health

Previous studies have shown a strong association between early motherhood (usually defined as a teenage birth) and higher mortality (Barclay et al. 2016; Doblhammer 2000; Grundy 2009; Grundy and Kravdal 2010; Grundy and Tomassini 2005; Henretta 2007; Mirowsky 2005) and higher risk of disability, long-term illness and poorer self-reported general health in mid and later life (Grundy and Read 2015; Grundy and Foverskov 2016; Grundy and Tomassini 2005; Henretta 2007; Read et al. 2011; Waldron et al. 1998). Some studies have also found later life mental health disadvantages for teenage mothers in some contexts (Grundy and Foverskov 2016; Spence 2008; Umberson et al. 2010), but others suggest these may reflect antecedent poorer mental health among women who become teenage mothers, and overall the evidence on early motherhood and depression is so far inconclusive. Physical health disadvantages for younger first-time fathers have also been reported (Einiö et al. 2015, 2016; Grundy and Kravdal 2010; Grundy and Read 2015; Read et al. 2011).

Potential explanations include social selection factors, possible direct influences (mainly for women), and possible indirect influences of fertility on subsequent life circumstances, which in turn affect later life health. First, those who become parents at young ages are more likely to come from deprived backgrounds, disrupted families, and have lower pre-parenthood education (Imamura et al. 2007; Kiernan 1997), factors associated with poorer later life health (Ploubidis et al. 2014). However, it is unlikely that social selection is a complete explanation, because the early parenthood-poor health association has also been observed in several studies which adjust for early life factors (Grundy and Kravdal 2007; Grundy and Foverskov 2016; Henretta 2007), or which use sibling designs to minimise confounding by family of origin (Barclay et al. 2016; Einiö et al. 2015, 2016). Early life poor health, which is strongly associated with childhood socio-economic status, may also be an important selection factor, as unhealthy individuals have poorer socio-economic trajectories and may marry others with similar poor health (Monden 2007). Assortative mating on health status and other social factors may, in turn, strengthen and amplify individual disadvantage.

Secondly, it is likely that the physiological processes of childbearing directly influence women’s risk of disease. Early or repeat experience of pregnancy, childbirth and breastfeeding are protective against breast, uterine and ovarian cancer (Grundy and Kravdal 2010; Merritt et al. 2015) due to reduced exposure to the hormones oestrogen and progesterone (Merrill et al. 2005). However, these advantages may be outweighed by social disadvantages, including poorer health related behaviours, associated with younger parenthood. Earlier first-time parents have higher mortality from smoking and alcohol-related causes, than those who became parents at a later age (Barclay et al. 2016; Grundy and Kravdal 2010), and a UK study found that associations between early parenthood and long-term illness were partially mediated by smoking and exercise (Grundy and Read 2015). Younger mothers may also have differential healthcare-seeking behaviour including poorer or delayed antenatal care (Downe et al. 2009). A further explanation is that early parenthood indirectly affects health through employment, wealth and education. Studies in the USA and UK show that earlier parenthood is associated with unstable employment trajectories (Dariotis et al. 2011; Sigle-Rushton 2005), less resource accumulation prior to childbearing (Powell et al. 2006) and lower educational attainment [although there is some debate over the direction of association with education (Kane et al. 2013)], which may lead to long-term health disadvantages (Dariotis et al. 2011; Lacey et al. 2017). Here, societal/welfare factors may play a role in moderating the negative health effects of early parenthood. A recent study found that early parenthood in Eastern Europe was not associated with poorer later life health compared with Western Europe, which may be explained by different normative effects and institutional supports for young parents (Grundy and Foverskov 2016).

### Parity and Later Life Health

Studies of developed countries typically find a J- or U-shaped association between parity and all-cause mortality, with childless, low or high parity (4, 5 or more children) having higher mortality than parents of 2 or 3 children (Barclay et al. 2016; Dior et al. 2013; Doblhammer 2000; Grundy 2009; Grundy and Tomassini 2005). Lifetime childlessness is associated with long-term illness (Grundy and Tomassini 2005), poorer midlife physical function for men (Guralnik et al. 2009), and poorer cognition (Read and Grundy 2017) in later life. Nulliparity is also associated with higher mortality from cancer, respiratory diseases and cardiovascular disease (Barclay et al. 2016; Einiö et al. 2016), suggesting that health behaviours such as smoking play a role, and that those who become parents either already had more healthy habits, or modified their health behaviours as parenthood provides greater social control and incentives to live healthily (Umberson et al. 2010). The evidence on associations between childlessness and depression is rather mixed (see for example, Buber and Engelhardt 2008; Hank 2010; Kravdal et al. 2017).

Perspectives from evolutionary biology such as ‘disposable soma theory’(Westendorp and Kirkwood 1998) and the theory of ‘maternal depletion’(Winkvist et al. 1992) suggest a trade-off between parity and longevity in women, with high parity associated with health disadvantage. Although not all studies find a parity-mortality penalty (Chereji et al. 2013; Grundy and Kravdal 2007; Hank 2010; Spence 2008), higher parity has been found to be associated with metabolic syndrome, obesity, diabetes and coronary heart disease (Gunderson et al. 2009; Hinkula et al. 2006; Lawlor et al. 2003), poorer self-rated health, health limitations (Read et al. 2011), and higher allostatic load and long-term illness (Grundy and Read 2015). Associations with metabolic risk factors persist after adjustment for lifestyle factors and socio-economic background (Umberson et al. 2011). It is thought that repeat pregnancy could lead to long-term lipid and glucose metabolism deficiencies (Skilton et al. 2009). On the other hand, higher parity is protective against uterine, breast and ovarian cancer (Barclay et al. 2016; Grundy and Kravdal 2010).

The role of broader biosocial influences, including long-term health behaviours (Grundy and Kravdal 2010), is indicated by the fact that similar, albeit smaller, associations between parenting history and health have been observed in men. For example, fatherhood is associated with permanent weight gain (Umberson et al. 2011), and in Norway both mothers and fathers of 4 or more children have lower mortality from alcohol-related causes (Grundy and Kravdal 2010). There are also multiple biosocial effects of large family size, for example, the accumulated stress of repeat childrearing, substantial economic costs and role overload could lead greater physical wear and tear, as indicated by high parity men and women having higher levels of allostatic load in later life (Grundy and Read 2015).

### Study Aims and Contribution

The aim of this study is to investigate associations between fertility history and changes in a range of health indicators measured over a 2–3 year period among adults 50–79 years: functional limitations, grip strength, depression, cognition, circulatory diseases, musculoskeletal diseases and cancer. Previous studies on this topic using Survey of Health, Ageing and Retirement in Europe (SHARE) have found somewhat mixed results for parenthood and depression: one found that parents of 2 children had reduced depression, compared with the childless (Hank and Wagner 2013); the other found no differences in depression by number of children (Gibney et al. 2017). However, the analyses were confined to one health outcome. Our health indicators include one well-established biomarker (grip strength), and validated scales for depression and cognitive function, which provide greater robustness than studies based on a few outcomes or relying on self-reported health alone. We also analyse prospective changes in health, and longitudinal studies on the topic (with the exception of mortality) are relatively sparse (Grundy and Read 2015; Read et al. 2011). In addition, we conduct analyses of interactions between fertility history and societal/welfare context (Grundy 2009; Grundy and Foverskov 2016), and fertility and marital status on later life health (Kravdal et al. 2012).

Based on previous research we expect to see that younger first-time parents (especially mothers) have worse outcomes on all indicators, including depression. As discussed above this is likely due to a combination of adverse selection factors, and possible ‘causal’ effects of early parenthood on life course outcomes, including health. We would also expect to see poorer health among the childless and those of high parity, but in different domains. Childless individuals, we hypothesise, will be more likely to have chronic diseases, especially those related to health behaviours, such as cancer and cardiovascular diseases, because they have not been subject to the social control influences of parenthood and are less likely to have been continuously partnered. In women, reproductive system cancer prevalence/incidence is likely to be higher in childless and lower parity individuals due to increased hormonal exposures. Higher parity mothers are more likely to have higher incidence of metabolic system diseases (related perhaps to weight gain), and to experience functional limitations and declines in muscular strength, reflecting the greater stress of repeated childbearing.

Based on previous research, we anticipate that fertility patterns may exert stronger effects on health in areas with weak welfare provision (in our study, Southern Europe), because in such societies the stresses and costs of childrearing fall to a greater extent on individuals and families. The effect of parity on health is likely conditioned by marital status—previous work suggests that the combination of parenthood with never being married carries a higher risk than other groups (Kravdal et al. 2012).

## Methods

### Data

We used individual-level data from waves 1–2 of SHARE (Börsch-Supan et al. 2013), a longitudinal cross-national survey representative of the non-institutionalised population aged 50 years and over. Wave 1 took place in 2004–2005, and wave 2 was conducted in 2006–2007. We used data from ten countries—Austria, Belgium, Denmark, France, Greece, Italy, the Netherlands, Spain, Sweden and Switzerland, excluding one country (Germany) because the individual follow-up rate from waves 1–2 was less than 60%. We excluded those aged 80+ because most SHARE surveys did not sample from institutions, and in some countries the proportion of over 80s in institutions reaches 20%; entry to which is associated with family size (Tomassini et al. 2004). After excluding participants who dropped out by wave 2 (either through loss to follow-up or death), and/or had no weights, our sample consisted of 6874 men and 8053 women. In further analysis we included individuals with missing data at follow-up which increased the sample size to 10,022 men and 11,555 women (details below).

### Measures

#### Health Outcomes

We use seven self-reported and objectively measured indicators of health collected at baseline (wave 1) and follow-up (wave 2). Functional limitations were measured using the 10-item Nagi scale and treated as an additive index. We created binary indicators for reporting having any doctor-diagnosed circulatory or metabolic disease (from a list including heart attack, stroke, hypertension, high blood cholesterol and diabetes), any doctor-diagnosed musculoskeletal disease (arthritis, including osteoarthritis or rheumatism, osteoporosis and hip or femoral fracture), and doctor-diagnosed cancer (excluding minor skin cancers). For women we separated reproductive cancers (uterine, ovarian and breast) and non-reproductive cancers, given that the literature suggests opposing effects. Dummy indicators were positive if the disease onset occurred after the respondent’s last child, to eliminate the possibility of chronic illness affecting fertility. Physical health was objectively assessed using grip strength, which is a strong prospective predictor of later life morbidity (Rantanen et al. 2000; Rantanen et al. 1999). Grip strength was measured using a handheld dynamometer and respondents provided multiple measurements with alternating hands (the highest value was used). Measurements of 0 or 100 kg or more, or those that differed by more than 20 kg were considered invalid and set to missing. Number of depressive symptoms was measured using the Euro-Depression (Euro-D) scale (0–12), validated for use in the European population (Castro-Costa et al. 2008). Cognition was assessed by a cognition index (combined scores of verbal fluency, immediate recall, delayed recall, orientation and numeracy). We also created an additive index of health problems (0–7), which comprised being in the bottom (within-gender) quintile of grip strength, bottom quintile of cognition, having > 6 depressive symptoms, at least one functional limitation and any of the chronic diseases.

#### Exposure: Fertility History (Reported at Wave 1)

Our parity indicator was based on reports of living biological children (0, 1, 2, 3 and 4 or more children). Considering only living, rather than ever-born children is problematic because of misclassifying those with deceased children. However, data on deceased children are only available in SHARELIFE (or wave 3 of the SHARE survey) which had a relatively high non-response rate. As a robustness check we repeated the analysis using a subset of respondents who took part in SHARELIFE and including an indicator of having a deceased child. Following other studies (Barclay et al. 2016; Grundy and Kravdal 2010) we used a categorical indicator of age at first birth which for women was grouped into < 20, 20–29 and 30 years or older, and for men was grouped < 23, 24–34 and 35 years or older.

#### Other Variables

We included several variables from wave 1 likely to be related to both fertility history and later life health. To control for socio-economic selection we included respondent’s father’s last occupation, standardised using the International Standard Classification of Occupations (ISCO) 1988 version. We dichotomised the variable into blue collar versus white collar where blue collar included codes from 6 upwards (skilled agricultural and fishery workers, craft and related tradesmen, plant/machine operators, elementary occupations and unemployed/not working). Respondent’s own education was standardised using the International Standard Classification of Education (ISCED) levels 1–6, and we used a three-category variable distinguishing those with low (levels 1–2), medium (3–4) and higher education (5–6). We used a measure of household wealth which summed the value of all owned assets, minus any debt, constructed within-country quintiles and used a binary variable of the bottom quintile versus the rest. Marital status was classified into never married, married or cohabiting, divorced and widowed. We included variables for age in years and age squared to account for the nonlinear decline in health with age. We also included (in sequential models) self-reported measures of current health behaviours at wave 1, because they are likely related to both fertility history and health. These were smoking (never/ex/current), and frequency of moderate/vigorous physical activity (more than once per week/once per week, 1 or 2 times per month, rarely or never). We created a variable for societal/welfare context by classifying countries into three groups: Nordic (Sweden, Denmark and the Netherlands), Continental (Belgium, France, Austria, Switzerland) and Mediterranean (Greece, Italy and Spain). We chose these groups based on broadly similar fertility trends over time (Frejka et al. 2008) and similar welfare policies (Arts and Gelissen 2002) over the decades when our cohorts were engaged in childrearing.

### Analytical Methods

To investigate change in health across two waves we used ‘conditional change score models’ where the outcome at follow-up *Y*_2_ is regressed on the earlier measure *Y*_1_ and a set of covariates *X.* This is mathematically equivalent to the ‘change score method’, where each individual has an individual score (*Y*_2_ − *Y*_1_) with the additional advantage that regression to the mean is less likely (Allison 1990). To adjust for differences in follow-up time, we included a variable indicating the number of months between surveys waves 1 and 2. Models included country fixed-effects to adjust for within-country homogeneity, and we computed robust standard errors adjusting for within-country clustering. We also calculated weighted estimates, using the calibrated cross-sectional and longitudinal weights provided with the SHARE data, which take account of study design, and weight to the known proportions of age and sex in each country at the start of the survey. The longitudinal weights additionally account for mortality by weighting to the population who survive to the end of the observation period. To model chronic diseases we used logistic regression. Number of functional limitations and depressive symptoms were modelled using negative binomial regression because of the large number of zeros in the distribution. Ordinal logistic regression was used for the index of poor health conditions. Grip strength and cognition score were approximately normally distributed so were modelled using ordinary least squares regression. Given that the hypothesised effects may operate differently by gender, we analysed men and women separately. We tested for significant interactions between fertility history and societal context, and fertility history and marital status using nested models and likelihood ratio tests. Any interactions significant at the 5% level were shown graphically using predicted probabilities.

It is well known that survey attrition in observational studies, if ignored, can lead to biased estimates of the relationships of interest. In our analysis, this is a particular problem since attrition between SHARE waves 1–2 ranged from 20% in Greece to 36% in France, and this is likely to be higher in those with worse health status. To assess whether our findings were affected by bias caused by missing data, we imputed missing covariates and responses using the multiple imputation by chained equations (MICE) approach in STATA 14.0. This assumes that the selection mechanism is ignorable under the missing at random (MAR) assumption (Rubin 1976), which states that the probability of missingness is entirely explained by the variables observed and included in the analysis model. The chained equation process continued for 20 cycles, which created 200 imputed datasets. The results of analyses were then combined using Rubin’s rules (Rubin and Little 1987). In the main text we present the MI results; the results using the complete case sample are available in the appendix. We also conducted sensitivity analysis using pattern mixture models (Daniels et al. 2011) to assess whether arbitrary assumptions about the missing data mechanism affected the results. We reran the analyses assuming that among those lost to follow-up the proportion in poor health was increased by 20 and 33%. All analyses were performed in STATA 14.0 (StataCorp 2015).

## Results

### Descriptive Statistics

**Fig. 1:**
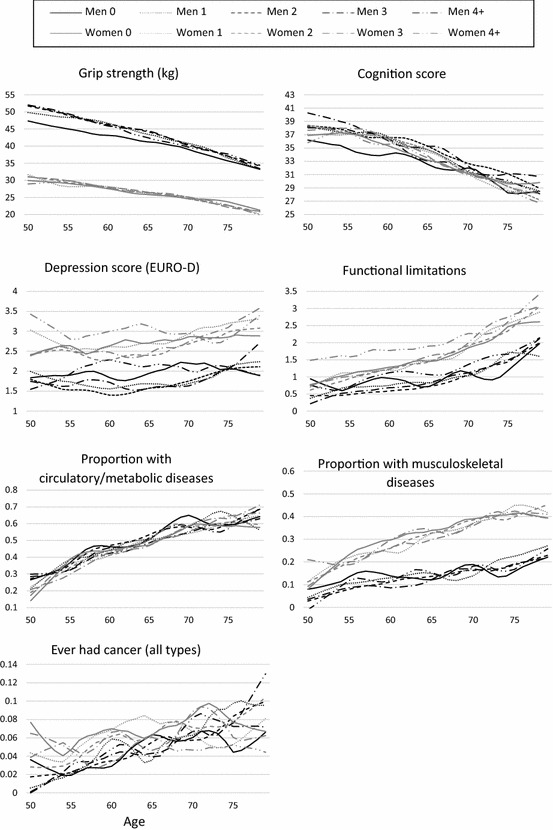
Study outcomes by age, gender and number of children, SHARE wave 1 [data smoothed using LOWESS (locally weighted scatterplot smoothing)]

**Fig. 2:**
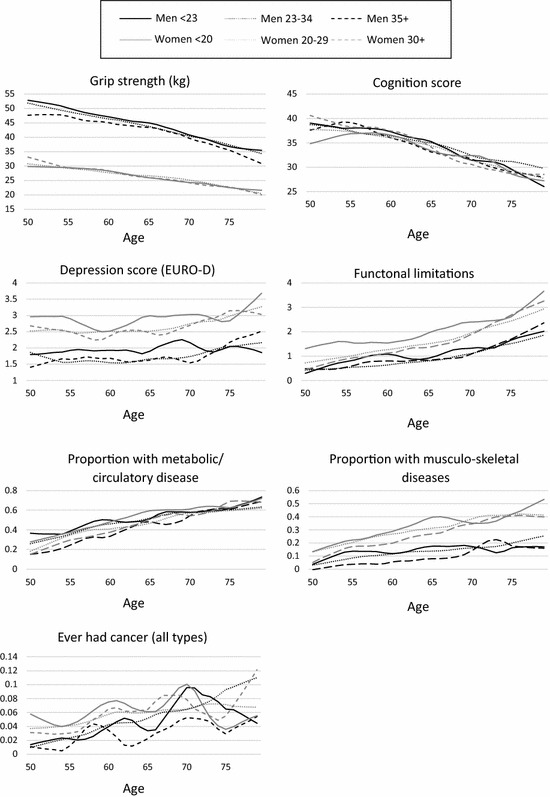
Study outcomes by age, gender and fertility timing, SHARE wave 1, parents only [data smoothed using LOWESS (locally weighted scatterplot smoothing)]

Table 1 shows descriptive statistics for all variables for all respondents, and for parents, present at waves 1 and 2. Eleven per cent of men and 10% of women were childless, and 11% of men and 15% of women had their first child at an early age. Women generally reported more health problems than men: they had more functional limitations, depressive symptoms and a higher prevalence of musculoskeletal diseases. There were less pronounced gender differences for circulatory diseases, cognition scores and cancer. Women were also more likely to have a lower level of education than men. Approximately one-third of the sample had a father with a ‘white-collar’ occupation. Women reported better health behaviours than men, with two-thirds reporting being never smokers, compared to approximately one-third of men. There were few gender differences in levels of reported physical activity. Figures 1 and 2 show the distribution of the health outcomes according to age, gender and fertility history. Health declined with age for all outcomes except depression. Women have higher depression, functional limitations, musculoskeletal diseases and lower grip strength over all ages. For many outcomes, younger mothers have a noticeable health disadvantage at age 50 (Fig. 2). Childless men had noticeably lower grip strength and cognition scores than other groups in the younger age bands (Fig. 1).

**Table 1:** Distribution of health and socio-demographic characteristics, SHARE wave 1, men and women aged 50–79 years (weighted complete case analysis)

Wave 1 characteristics	All men	Fathers	All women	Mothers
	*N* (%)	*N (*%)	*N (*%)	*N* (%)
Fertility characteristics^a^				
Number of children				
0	10.6		9.9	**–**
1	15.1	16.9	17.1	19.0
2	40.8	45.6	39.0	43.3
3	20.1	22.4	20.2	22.4
4+	13.5	15.1	13.8	15.3
AFB				
< 20/< 23 years		11.0		14.7
20–29/23–34 years		78.6		70.1
30+/35+		10.5		15.1
Health outcomes				
Functional limitations, mean (SD)	0.81 (1.53)	0.81 (1.50)	1.53 (2.06)	1.53 (2.06)
Circulatory and metabolic diseases	47.7	47.6	45.7	45.8
1 + circulatory/metabolic disease by age 70	44.7	44.7	42.9	43.1
Musculoskeletal diseases	12.5	12.4	29.3	29.6
1 + musculoskeletal disease by age 70	11.7	11.6	28.6	28.2
Cancers	3.9	3.9	2.0	2.1
1 + cancer by age 70	3.3	3.3	4.9	4.8
Reproductive system cancers	–	–	3.5	3.3
Grip strength (kg), mean (SD)	44.5 (9.8)	44.7 (9.8)	27.1 (6.7)	27.1 (6.9)
Cognition score, mean (SD)	35.3 (9.6)	35.4 (9.6)	34.9 (9.9)	35.0 (9.9)
Depressive symptoms, mean (SD)	1.7 (1.9)	1.7 (1.9)	2.7 (2.3)	2.7 (2.3)
Index of health problems, mean (SD)	1.4 (1.3)	1.4 (1.3)	2.0 (1.5)	2.0 (1.5)
Health behaviours				
Smoking: never	2324 (33.9)	2040 (33.3)	5215 (64.8)	4719 (65.1)
Ex-smoker	2855 (41.6)	2631 (42.9)	1479 (18.4)	1343 (18.5)
Current smoker	1682 (24.5)	1462 (23.8)	1349 (16.8)	1189 (16.4)
Moderate/vigorous physical activity > more once per week	79.0	79.3	76.1	76.5
Age, mean (SD)^a^	62.8 (8.1)	62.8 (8.1)	62.6 (8.1)	62.6 (8.1)
Education				
Low (ISCED 1–2)	48.4	47.9	57.9	58.7
Medium (ISCED 3–4)	29.7	30.2	25.5	25.4
High (ISCED 5–6)	21.9	21.9	16.7	16.0
Father’s occupation: white collar	33.0	33.2	33.1	32.6
Blue collar/not working	67.0	66.8	66.9	67.4
Marital status				
Never married	5.8	1.4	5.3	1.4
Married/partnered	83.9	72.2	68.7	72.3
Divorced	5.7	7.7	7.7	7.7
Widowed	4.6	18.7	18.2	18.6
Welfare state group				
Continental	38.4	38.5	38.2	38.1
Nordic	30.7	29.4	31.1	29.9
Mediterranean	30.9	32.1	30.8	32.0
Total *N*	6874	6144	8053	7258

^a^Fertility characteristics and age values were taken from wave 2 responses if wave 1 were missing

### Cross-Sectional Results

**Fig. 3:**
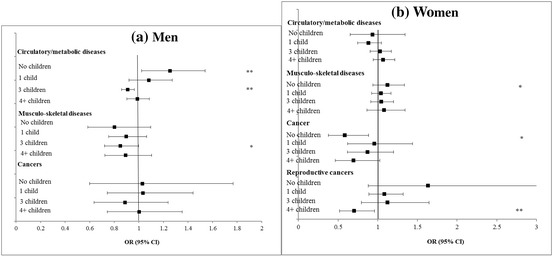
**a**, **b** Adjusted cross-sectional associations between fertility history and chronic diseases at baseline among men and women aged 50–79 years, SHARE wave 1. *P < 0.05 **P < 0.01 ***P < 0.001

**Fig. 4:**
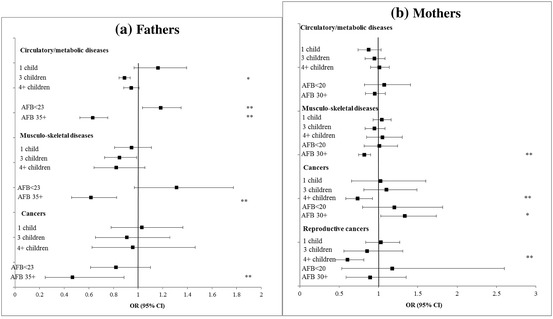
**a**, **b** Adjusted cross-sectional associations between fertility history and chronic diseases at baseline among parents aged 50–79 years, SHARE wave 1. *P < 0.05 **P < 0.01 ***P < 0.001

Tables 2, 3 and Figs. 3 and 4 show the cross-sectional associations between fertility history and health indicators at baseline (wave 1), with Table 2 showing the models for functional limitations with the full covariates. Table 2 shows that men and women who had 4 or more children (compared with two) had at least 20% higher odds of functional limitations. Teenage mothers had more functional limitations, compared with the reference group who had had their first child at age 20–29 years. The covariate coefficients show the expected socio-demographic associations with health: higher prevalence of health problems among older people, and those with lower education and household wealth. Being an ex-smoker or doing less physical activity was associated with more functional limitations. There were significant differences in functional limitations by country, with Greece, Spain and Austria having the highest odds (compared with Belgium) and Switzerland the lowest.

**Table 2:** Adjusted cross-sectional associations between index of functional limitations and fertility history, showing full covariates, SHARE wave 1, men and women aged 50–79 years (multiple imputation, weighted data)

Index of functional limitations	All men IRR (95% CI)	Fathers IRR (95% CI)	All women IRR (95% CI)	Mothers IRR (95% CI)
Fertility characteristics				
Number of children (ref: 2)				
0	1.07 (0.96–1.19)		0.96 (0.89–1.03)	
1	0.97 (0.85–1.11)	0.96 (0.83–1.11)	**1.08 (1.01–1.16)**	**1.09 (1.01–1.17)**
3	1.04 (0.98–1.10)	1.03 (0.97–1.09)	**1.07 (1.02–1.12)**	**1.06 (1.01–1.11)**
4+	**1.24 (1.13–1.36)**	**1.22 (1.10–1.36)**	**1.29 (1.23–1.35)**	**1.25 (1.19–1.32)**
AFB (ref: 20–29/23–34 years)				
< 20/< 23 years		1.08 (0.83–1.41)		**1.14 (1.05–1.23)**
30+/35+		1.06 (0.98–1.14)		0.95 (0.89–1.01)
Other covariates				
Age (continuous)	**1.04 (1.04–1.05)**	**1.04 (1.03–1.05)**	**1.04 (1.03–1.04)**	**1.04 (1.03–1.04)**
Age squared	1.00 (0.99–1.00)	1.00 (0.99–1.00)	0.99 (0.99–0.99)	1.00 (0.99–1.00)
Education: (ref: low ISCED 1–2)				
Medium (ISCED 3–4)	**0.71 (0.63–0.81)**	**0.74 (0.68–0.81)**	**0.82 (0.73–0.92)**	**0.81 (0.71–0.93)**
High (ISCED 5–6)	**0.54 (0.46–0.63)**	**0.59 (0.51–0.68)**	**0.65 (0.53–0.79)**	**0.66 (0.55–0.80)**
Father’s occupation: blue collar/not working	1.02 (0.92–1.14)	1.04 (0.93–1.17)	1.02 (0.92–1.12)	1.04 (0.96–1.13)
Low household wealth	**1.34 (1.14–1.57)**	**1.46 (1.33–1.60)**	0.94 (0.88–1.01)	**1.37 (1.27–1.47)**
Smoking: (ref: never)				
Ex-smoker	**1.44 (1.33–1.57)**	**1.33 (1.16–1.51)**	**1.10 (1.04–1.18)**	**1.09 (1.03–1.15)**
Current smoker	**1.24 (1.14–1.36)**	**1.19 (1.04–1.36)**	1.02 (0.94–1.10)	1.02 (0.94–1.11)
Frequency of moderate/vigorous physical activity, ordinal (> once per week to hardly ever)	**1.49 (1.42–1.57)**	**1.57 (1.48–1.67)**	**1.33 (1.28–1.38)**	**1.31 (1.26–1.37)**
Marital status (ref: married/partnered)				
Never married	1.06 (0.86–1.30)	1.27 (0.97–1.66)	0.90 (0.79–1.03)	1.08 (0.97–1.21)
Divorced	1.20 (0.96–1.50)	1.03 (0.87–1.23)	1.07 (0.94–1.21)	1.09 (0.90–1.32)
Widowed	1.03 (0.96–1.11)	1.04 (0.88–1.23)	0.93 (0.81–1.08)	0.91 (0.80–1.05)
Country (ref: Belgium)				
Austria	**1.32 (1.19–1.46)**	**1.23 (1.12–1.35)**	**1.23 (1.12–1.35)**	1.05 (0.98–1.11)
Denmark	**0.87 (0.78–0.97)**	0.91 (0.82–1.01)	0.91 (0.82–1.01)	0.97 (0.91–1.03)
France	**0.90 (0.86–0.94)**	**0.94 (0.91–0.97)**	**0.94 (0.91–0.97)**	**0.95 (0.92–0.97)**
Greece	0.95 (0.82–1.10)	0.95 (0.87–1.05)	0.95 (0.87–1.05)	**1.19 (1.09–1.31)**
Italy	**0.77 (0.67–0.89)**	**0.78 (0.70–0.86)**	**0.78 (0.70–0.86)**	0.99 (0.90–1.10)
Netherlands	**0.79 (0.68–0.91)**	**0.80 (0.71–0.89)**	**0.80 (0.71–0.89)**	**0.88 (0.82–0.96)**
Spain	1.02 (0.89–1.16)	**1.16 (1.04–1.29)**	**1.16 (1.04–1.29)**	**1.27 (1.13–1.42)**
Sweden	**0.69 (0.62–0.78)**	**0.72 (0.66–0.80)**	**0.72 (0.66–0.80)**	**0.95 (0.89–1.00)**
Switzerland	**0.59 (0.51–0.67)**	**0.58 (0.50–0.66)**	**0.66 (0.59–0.73)**	**0.67 (0.62–0.73)**
Total *N*	9805	8902	11,439	10,374

Bold indicates statistically significant at the 5% level

**Table 3:** Adjusted cross-sectional associations between fertility history and health outcomes at baseline among men and women aged 50–79 years, SHARE wave 1

	Grip strength *β* (95% CI)	Depression IRR (95% CI)	Cognition *β* (95% CI)	Index of health conditions OR (95% CI)
All men (*N* = 9805)				
No. children (ref: 2)				
0	− 1.07 (− 2.50–0.35)	0.92 (0.77–1.09)	− 0.74 (− 1.52–0.04)	0.97 (0.77–1.22)
1	− 0.25 (− 0.72–0.22)	0.98 (0.88–1.09)	− 0.04 (− 0.80–0.72)	0.98 (0.84–1.13)
3	− 0.54 (− 1.69–0.61)	**1.05 (1.00–1.10)**	− 0.12 (− 0.52–0.28)	0.89 (0.77–1.04)
4+	− 0.65 (− 2.33–1.03)	**1.12 (1.02–1.24)**	− **1.36 (**− **2.71–0.01)**	**1.20 (1.05–1.37)**
Fathers (*N* = 8902)				
No. children (ref: 2)				
1	0.07 (− 0.43–0.57)	0.96 (0.85–1.09)	− 0.01 (− 0.81–0.78)	0.97 (0.81–1.17)
3	− 0.59 (− 1.79–0.62)	**1.05 (1.00–1.10)**	− 0.18 (− 0.59–0.23)	1.04 (0.94–1.15)
4+	− 0.75 (− 2.48–0.97)	**1.12 (1.02–1.23)**	− **1.45 (**− **2.84−**− **0.06)**	**1.28 (1.06–1.54)**
AFB (ref: 23–34 years)				
< 23 years	− 0.4 (− 1.57–0.77)	1.06 (0.96–1.17)	− 0.01 (− 1.57–1.55)	1.18 (0.91–1.54)
35+	− **1.47 (**− **2.47–0.48)**	1.06 (0.90–1.26)	− **0.58 (**− **1.06−**−** 0.10)**	0.89 (0.77–1.04)
All women (*N* = 11,439)				
No. children (ref: 2)				
0	0.16 (− 1.05–1.38)	0.95 (0.89–1.02)	− **0.79 (**− **1.53–**−** 0.05)**	0.90 (0.77–1.06)
1	− 0.43 (− 1.07–0.21)	**1.07 (1.00–1.14)**	− 0.56 (− 1.12–0.01)	1.04 (0.91–1.19)
3	0.03 (− 0.46–0.51)	1.00 (0.95–1.06)	− 0.35 (− 0.80–0.09)	1.00 (0.90–1.10)
4+	− 0.15 (− 0.90–0.59)	1.05 (0.99–1.10)	− **0.92 (**− **1.64–**− **0.21**)	**1.25 (1.06–1.48)**
Mothers (*N* = 10,374)				
No. children (ref: 2)				
1	− 0.49 (− 1.06–0.07)	**1.06 (1.00–1.13)**	− 0.54 (− 1.15–0.07)	1.07 (0.97–1.18)
3	0.12 (− 0.35–0.58)	1.01 (0.95–1.06)	− 0.31 (− 0.82–0.19)	1.01 (0.92–1.11)
4+	− 0.07 (− 0.81–0.68)	**1.05 (1.00–1.09)**	− **0.88 (**− **1.66–**− **0.10)**	**1.22 (1.11–1.34)**
AFB (ref: 20–29 years)				
< 20 years	− 0.19 (− 0.59–0.22)	1.00 (0.96–1.05)	− 0.15 (− 0.87–0.57)	1.03 (0.81–1.31)
30+	0.30 (− 0.23–0.84)	1.01 (0.97–1.05)	0.09 (− 0.65–0.83)	0.91 (0.82–1.01)

Adjusted for age (continuous), age squared, educational level, father’s occupation, wealth quintile, smoking, reported physical activity, marital status and country fixed-effects

Bold indicates statistically significant at the 5% level

Turning to the cross-sectional results shown in Table 3 and Figs. 3 and 4 for specific dimensions of health and chronic diseases, later first-time fathers had poorer cognitive function and lower grip strength, but reduced odds of metabolic and musculoskeletal diseases. Fathers of 4 or more children had more depressive symptoms, worse overall health and lower cognition. Men who became fathers before the age of 23, and childless men had increased likelihood of having circulatory/metabolic diseases. Among women, high parity (4+) was associated with lower cognition, more health problems, but lower risk of reproductive cancers compared to mothers of 2 children. Becoming a mother at age 30 or over was associated with a lower risk of musculoskeletal diseases. Childless women had lower risks of non-reproductive cancers but lower cognition scores. Associations with depression were slight.

### Longitudinal Associations Between Fertility History and Health

**Fig. 5:**
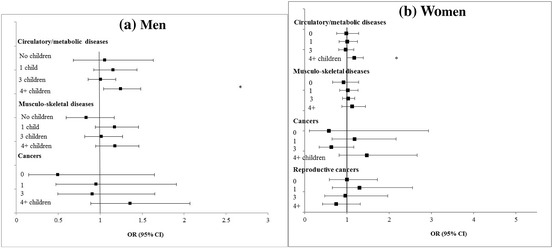
**a**, **b** Adjusted longitudinal associations between fertility history and chronic diseases in men and women aged 50–79 years, SHARE waves 1–2. *P < 0.05 **P < 0.01 ***P < 0.001

**Fig. 6:**
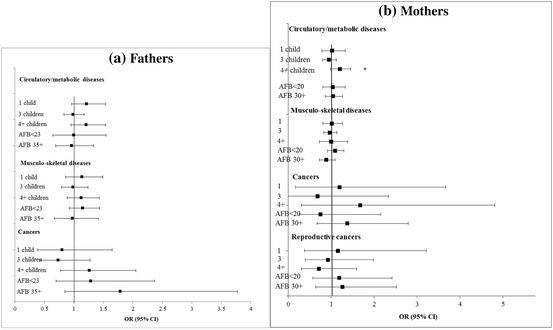
**a**, **b** Adjusted longitudinal associations between fertility history and chronic diseases in parents aged 50–79 years, SHARE waves 1–2. *P < 0.05 **P < 0.01 ***P < 0.001

Table 4 and Figs. 5 and 6 show changes in health between waves 1 and 2 according to fertility history. Parents of 4 or more children had a higher risk of circulatory/metabolic disease onset. Overall, women who had been teenage mothers had a higher risk of increasing health problems and functional limitations. Parents of 4 or more children had higher odds of developing metabolic diseases (Fig. 5a, b). We undertook additional regressions, sequentially adding physical activity and smoking behaviour at baseline, but this hardly altered the results.

**Table 4:** Adjusted longitudinal associations between fertility history and health outcomes at follow-up (wave 2) among men aged 50–79 years, SHARE waves 1–2

	Grip strength β (95% CI)	Functional limitations IRR (95% CI)	Depression IRR (95% CI)	Cognition β (95% CI)	Index of health conditions OR (95% CI)
All men (*N* = 9805)					
No. children (ref: 2)					
0	− 0.35 (− 2.01–1.31)	1.10 (0.85–1.42)	1.13 (0.95–1.35)	0.07 (− 1.40–1.54)	1.06 (0.84–1.34)
1	− 0.06 (− 0.91–0.78)	1.05 (0.89–1.25)	1.09 (0.95–1.25)	0.09 (− 0.88–1.07)	1.15 (0.83–1.59)
3	− 0.48 (− 1.34–0.39)	1.07 (0.96–1.19)	**1.07 (1.00–1.15)**	− 0.04 (− 1.25–1.17)	1.16 (0.95–1.41)
4+	− 0.61 (− 1.67–0.45)	1.07 (0.94–1.21)	1.06 (0.99–1.14)	− 0.21 (− 1.25–0.83)	1.16 (0.96–1.40)
Fathers (*N* = 8902)					
No. children (ref: 2)					
1	− 0.14 (− 0.96–0.68)	1.04 (0.85–1.28)	1.11 (0.98–1.25)	− 0.07 (− 1.46–1.32)	1.15 (0.84–1.56)
3	− 0.42 (− 1.35–0.50)	1.04 (0.95–1.15)	1.07 (0.97–1.19)	− 0.08 (− 1.26–1.11)	1.13 (0.93–1.38)
4+	− 0.68 (− 1.68–0.32)	1.02 (0.92–1.13)	1.06 (0.94–1.18)	− 0.33 (− 1.36–0.70)	1.14 (0.98–1.33)
AFB (ref: 23–34 yrs)					
< 23 years	− 0.45 (− 1.39–0.48)	1.16 (0.92–1.46)	1.04 (0.92–1.17)	− 0.31 (− 1.45–0.83)	1.13 (0.92–1.39)
35+	0.14 (− 1.11–1.39)	0.94 (0.78–1.14)	0.94 (0.86–1.02)	− 0.19 (− 1.18–0.79)	0.98 (0.80–1.20)
All women (*N* = 11,439)					
No. children (ref:2)					
0	0.02 (− 1.00–1.04)	0.97 (0.87–1.08)	1.03 (0.95–1.12)	0.22 (− 0.96–1.39)	0.89 (0.73–1.09)
1	0.12 (− 0.58–0.82)	1.01 (0.94–1.09)	1.02 (0.94–1.12)	–0.1 (− 0.93–0.74)	1.00 (0.86–1.16)
3	− 0.18 (− 0.69–0.33)	0.94 (0.87–1.01)	1.00 (0.93–1.08)	–0.4 (− 1.05–0.25)	1.03 (0.89–1.20)
4+	− 0.25 (− 1.12–0.62)	1.03 (0.93–1.15)	1.04 (0.98–1.1)	− 0.63 (− 1.63–0.37)	**1.17 (1.05–1.30)**
Mothers (*N* = 10,374)					
No. children (ref:2)					
1	0.27 (− 0.41–0.94)	1.04 (0.97–1.12)	1.03 (0.96–1.11)	− 0.09 (− 0.70–0.52)	1.04 (0.89–1.22)
3	− 0.06 (− 0.75–0.62)	0.94 (0.87–1.02)	1.01 (0.94–1.08)	− 0.39 (− 1.10–0.31)	1.01 (0.86–1.19)
4+	− 0.04 (− 0.90–0.83)	1.02 (0.93–1.13)	**1.05 (1.00–1.09)**	− 0.61 (− 1.38–0.16)	**1.15 (0.99–1.33)**
AFB (ref: 20–29 yrs)					
< 20 years	0.13 (− 0.89–1.15)	**1.12 (1.03–1.22)**	1.05 (0.98–1.13)	0.01 (− 0.74–0.77)	**1.23 (1.04–1.45)**
30+	− 0.03 (− 0.82–0.76)	1.00 (0.90–1.11)	0.99 (0.93–1.06)	0.06 (− 0.89–1.01)	0.95 (0.79–1.15)

Adjusted for: health at baseline, months between wave 1 and wave 2, age (continuous), age squared, country fixed-effects, father’s occupation, education, marital status, parity (parent’s models), smoking behaviour, physical activity, household wealth. Depression additionally adjusted for physical health (functional limitations)

Bold indicates statistically significant at the 5% level

### Robustness and Sensitivity Analysis

The supplementary Tables S1–S3 and Figures S1–S4 show the same models with a weighted complete case analysis. There were more statistically significant associations in the complete case analyses, but the same pattern of effects was seen. Adjustment for deceased children (Table S4, cross-sectional models and Table S5, longitudinal models) did not substantially alter the results. Appendix Tables S6 and S7 show the results of pattern mixture models which assess the difference in the longitudinal results when the dropouts are assumed to be in worse health at baseline than those who remained. In the majority of cases the same pattern of associations held, the main exception was that the association with cancer in high parity women disappeared: the OR for women with 4+ children decreased from 2.31 to 1.09. This means that in general, our assumption that the data were missing at random using the MI results was appropriate, and that the results from the MI models are relatively robust.

### Interactions with Societal/Welfare Context and Marital Status

We found evidence that the effects of parity on overall health at baseline varied by context for both men and women (LLT *P* value for men *P* = 0.028, and for women *P* = 0.014). Men in Nordic countries with 4+ children fared better than parity 4+ men in other contexts. Women with 2 or more children in Mediterranean countries fared worse than women in the other countries, and the Mediterranean disadvantage increased with parity. We show the predicted probabilities in supplementary Figures S5a and S5b. We also saw that women’s grip strength declined with increasing parity in the Mediterranean countries but not in the other regions. We did observe some weakly significant interactions for fertility timing, but most of these showed no clear trend. We found some evidence that men with a high parity (3 or more) had worse overall health at baseline if they were also never married, compared to other marital status groups (LLT *P* value = 0.047). There was also the same significant interaction effect in the longitudinal models (Supplementary Figures S6a and S6b). We also found some evidence of parenthood timing was moderated by marital status, but there was no clear pattern to these differences.

## Discussion

In this longitudinal study following European respondents over a 2–3-year period, we found fertility patterns predicted greater increases in some health problems for older people, and differently for men and women. Our results confirm previous studies in finding that higher parity (4 or more children) and early parenthood is associated with poorer health and faster health declines. By investigating specific health indicators across a range of diseases and conditions using the same sample, we have gained a better understanding of underlying biosocial pathways. The negative effect of early parenthood was most evident through more baseline functional limitations and faster acquisition of limitations during the follow-up period, corroborating associations between early parenthood and poorer physical function seen in other studies (Barban 2013; Berkman et al. 2015; Grundy and Tomassini 2005; Hank 2010; Henretta et al. 2008; Pirkle et al. 2014). This pattern could be explained by health or social selection, or that early parenthood is part of a process of ‘weathering’, a chain of disadvantage through adolescence and adulthood which creates stress and cumulates in worse health (Foster et al. 2008; Geronimus 1996). Parents of 4 or more children had worse health at baseline on a range of measures: depression, cognition and circulatory disease. High parity parents were also more likely to develop circulatory disease during follow-up (in line with other studies (Lawlor et al. 2003), suggesting a potential biological influence of repeat childbearing (women) through metabolic and lipid profiles, or through lifestyle factors (both men and women). Later first-time fathers (age 35+) had lower grip strength and cognition at baseline, but also fewer chronic diseases, and did not experience significantly worse health declines. Although childless individuals had worse baseline health on some measures, they also did not experience greater health declines.

While the study largely confirms patterns seen previously, we did find some unexpected results and extended our understanding of the fertility–health nexus in different contexts. Adjustment for current health behaviours did not alter the results, suggesting they do not play a major explanatory role. This was surprising for smoking, because this also took into account former smoking. However, health behaviours through the life course could still be an important explanation, and future studies could address this. To fully elucidate this influence in future research it would be necessary to use prospective data covering the whole life course. A previous study has noted the absence of a parity penalty in Norway relative to other contexts (Grundy 2009), while a Swedish analysis found elevated mortality risks for parity 4+ men, but not for women, after controlling for fertility timing (Barclay et al. 2016). Our study found no high parity penalty in Nordic welfare regimes relative to others. This could be explained by greater institutional supports in Nordic regimes for women’s labour force participation, the availability of childcare and generous family benefits, which may offset potential stresses and costs of childrearing. We found some moderation of fertility patterns by current marital status, mainly that the combination of being never married and having 3 or more children was associated with worse health for men, than other partnership/fertility patterns. However, this analysis was limited by a lack of data on partnership history in the waves 1 and 2 SHARE data. Further research could start to investigate how fertility affects health conditional on life course partnership history, to unpick the complex pathways to poor health in later life. This could include accounting for partner’s characteristics and how these can influence later life health.

The later life health effects of childbearing postponement on parents are still not fully understood (Schmidt et al. 2012), partly because there is a lag between fertility and later life outcomes. Another complicating factor is that the social profile of those who give birth at advanced ages has changed over the century, altering associations with health: a recent UK study showed that across cohorts older mothers have become progressively more advantaged (Goisis et al. 2017). In this study, later first-time fathers (age 35+, born in the years 1961–1990) had significantly worse health on some measures, and significantly better on others. This could be explained by prior health conditions delaying entry to partnership and childbearing (Nilsen et al. 2013).On the other hand, later first-time fathers could also be actively parenting into middle age which could provide some social control of health related behaviours and more social participation, lowering the risk of developing lifestyle related diseases. An additional explanation is that later first-time parents are positively selected, considering themselves in good enough health to consider childbearing at later ages. However, the apparently contradictory results for later parenthood deserve further exploration, especially because childbearing postponement in increasing in most European countries.

Strengths of this study include the use of multiple health indicators (including one objectively measured biomarker) covering many health domains, use of a prospective design, and cross-national comparison of trends. We also paid attention to missing data mechanisms and performed substantial sensitivity analyses. However, there were several limitations. We followed individual’s health for a relatively short period of time (2–3 years) and levels of change were not dramatic. Following SHARE respondents over several waves is complicated by high levels of attrition (closely related to health status) which would result in an extremely unrepresentative sample. Despite applying weights that adjust to mortality over the observation period, because we only start observing our sample at age 50, there is likely to be survivorship bias at different levels for different countries. Although we included some early life variables in the model which might account for selection (education, father’s occupation) we lacked data on measures of early life health, cognition and health behaviours which may confound some of the associations seen in this study, although they are known to be strongly associated with socio-economic background and educational attainment which we do consider (Grundy and Read 2015). Our assessment of the impact of health behaviours was limited because we did not have data on life course health behaviours, and it would be important to look these effects dynamically. In addition we are testing for effects across multiple outcomes, which increases the chance of spurious ‘false positive’ results. In this respect it is reassuring that we broadly see the same pattern of effects across multiple specifications of the same model, while using weighted/unweighted data, multiply imputed data and other subsamples. Our study consolidates the evidence for fertility history affecting a range of range of health domains in later life, differently for men and women, and suggests further attention be paid to the mechanisms for poor health, including health behaviours, healthcare-seeking behaviour and interactions between fertility, marriage and socio-economic factors.

## Electronic Supplementary Material

Below is the link to the electronic supplementary material.

Supplementary material 1 (DOCX 337 kb)
