# Supplementary material for: Fertility History and Physical and Mental Health Changes in European Older Adults
Source: Eur J Popul. 2018 Apr 26;35(3):459–85. doi: 10.1007/s10680-018-9489-x (PMC6639520; doi:10.1007/s10680-018-9489-x)
Supplement: Supplementary file 1 — Supplementary material 1 (DOCX 337 kb) [file 10680_2018_9489_MOESM1_ESM.docx]

**Supplementary Material**

**Table S1: Adjusted cross-sectional associations between index of functional limitations and fertility history, showing full covariates, SHARE wave 1, men and women aged 50-79 years (complete case, weighted)**

| **Index of functional limitations** | All men  IRR (95% CI) | Fathers  IRR (95% CI) | All Women  IRR (95% CI) | Mothers  IRR (95% CI) |
| --- | --- | --- | --- | --- |
|  |  |  |  |  |
|  |  |  |  |  |
| *Fertility characteristics* |  |  |  |  |
| Number of children (ref: 2) |  |  |  |  |
| 0 | 1.03(0.86-1.23) |  | 1.04(0.97-1.12) |  |
| 1 | 1.06(0.9-1.25) | 1.03(0.88-1.22) | 1.12(0.99-1.26) | 1.10(0.98-1.23) |
| 3 | 1.03(0.91-1.18) | 1.03(0.91-1.17) | **1.11(1.04-1.18)** | **1.10(1.04-1.18)** |
| 4+ | 1.10(0.93-1.29) | 1.07(0.91-1.26) | **1.29(1.13-1.46)** | **1.27(1.11-1.44)** |
| AFB (ref: 20-29/23-34 years) |  |  |  |  |
| <20/<23 years |  | 1.10(0.86-1.41) |  | **1.13(1.01-1.25)** |
| 30+/35+ |  | **1.12(1.03-1.22)** |  | 1.06(0.99-1.13) |
| *Other covariates* |  |  |  |  |
| Age (continuous) | **1.04(1.03-1.05)** | **1.04(1.03-1.05)** | **1.03(1.03-1.04)** | **1.04(1.03-1.04)** |
| Age squared | 1.00 (0.99-1.00) | 1.00 (0.99-1.00) | 1.00 (0.99-1.00) | **0.99 (0.99-0.99)** |
| Education: (ref: low ISCED 1-2) |  |  |  |  |
| Medium (ISCED 3-4) |  | **0.62(0.5-0.77)** | **0.82(0.73-0.91)** | **0.8(0.71-0.92)** |
| High (ISCED 5-6) |  | **0.6(0.49-0.75)** | **0.65(0.57-0.74)** | **0.65(0.58-0.74)** |
| Father’s occupation:  blue collar/not working | 1.07(0.94-1.22) | 1.1(0.99-1.24) | 1.07(0.99-1.17) | **1.09(1.01-1.17)** |
| Low household wealth | **1.33(1.12-1.58)** | **1.31(1.05-1.63)** | **1.38(1.27-1.49)** | **1.38(1.28-1.48)** |
| Smoking: (ref: never) |  |  |  |  |
| Ex-smoker | **1.39(1.26-1.52)** | **1.38(1.25-1.51)** | **1.11(1.03-1.19)** | 1.1(0.98-1.24) |
| Current smoker | **1.25(1.09-1.43)** | **1.22(1.01-1.47)** | 0.92(0.81-1.04) | 0.94(0.86-1.02) |
| Frequency of moderate/vigorous physical activity, ordinal (>once per week to hardly ever) | **1.49(1.43-1.56)** | **1.52(1.48-1.56)** | **1.34(1.28-1.4)** | **1.32(1.26-1.39)** |
| Marital status (ref: married/partnered) |  |  |  |  |
| Never married | 0.94(0.83-1.07) | **1.49(1.17-1.88)** | 0.9(0.63-1.3) | 1.11(0.81-1.52) |
| Divorced | **1.45(1.1-1.93)** | **1.55(1.11-2.18)** | **1.13(1.01-1.27)** | 1.16(0.98-1.38) |
| Widowed | 0.95(0.8-1.11) | 0.93(0.78-1.11) | 0.94(0.82-1.08) | 0.92(0.8-1.06) |
| Country (ref: Sweden) |  |  |  |  |
| Austria | **1.4(1.3-1.5)** | **1.42(1.34-1.51)** | **1.04(1-1.08)** | 1.03(0.99-1.06) |
| Denmark | **0.88(0.8-0.96)** | 0.94(0.86-1.02) | 1.02(0.98-1.06) | 1.02(0.97-1.07) |
| France | **0.76(0.74-0.78)** | **0.75(0.74-0.76)** | 0.95(0.92-0.98) | **0.98(0.95-1)** |
| Greece | **1.07(1.02-1.11)** | **1.05(1.02-1.09)** | 1**.25(1.2-1.31)** | **1.27(1.2-1.34)** |
| Italy | **0.86(0.81-0.91)** | **0.89(0.84-0.94)** | 1.01(0.97-1.06) | **1.07(1.01-1.13)** |
| Netherlands | **0.86(0.83-0.89)** | **0.88(0.86-0.9)** | 0.97(0.94-0.99) | 0.96(0.93-0.99) |
| Spain | **1.11(1.05-1.18)** | **1.17(1.1-1.24)** | **1.37(1.28-1.47)** | **1.44(1.33-1.55)** |
| TOTAL N | 6123 | 5453 | 7281 | 6557 |

Bold indicates significant at 5% level.

**Table S2: Adjusted cross-sectional associations between fertility history and health outcomes at baseline among men and women aged 50-79 years, SHARE wave 1(complete case, weighted)**

| **ALL MEN** | **Grip strength**  **β(95% CI)** | **Depression**  **IRR (95% CI)** | **Cognition**  **β( (95% CI)** | **Index of health conditions**  **OR (95% CI)** |
| --- | --- | --- | --- | --- |
| **No. children (ref:2)** |  |  |  |  |
| 0 | -0.71(-2.95-1.54) | 0.93(0.76-1.14) | 0.08(-1.13-1.29) | 1.01(0.80-1.29) |
| 1 | -0.32(-1.55-0.91) | 1.00(0.95-1.05) | 0.15(-0.23-0.53) | 1.04(0.98-1.10) |
| 3 | -0.86(-2.16-0.44) | **1.06(1.04-1.09)** | -0.25(-0.70-0.20) | 0.97(0.89-1.06) |
| 4+ | -0.86(-2.71-0.99) | 1.08(0.97-1.21) | **-1.11(-2.21--0.01)** | **1.28(1.05-1.56)** |
| **FATHERS** |  |  |  |  |
| **No. children (ref:2)** |  |  |  |  |
| 1 | 0.11(-1.09-1.30) | 0.97(0.93-1.02) | 0.39(-0.03-0.81) | 1.02(0.95-1.10) |
| 3 | -0.94(-2.27-0.39) | **1.07(1.04-1.11)** | -0.28(-0.75-0.18) | 0.96(0.88-1.04) |
| 4+ | -0.90(-2.67-0.87) | 1.09(0.98-1.21) | **-1.09(-2.36-0.18)** | **1.22(1.00-1.49)** |
| **AFB (ref:** 23-34 years) |  |  |  |  |
| <23 years | -0.13(-1.70-1.44) | 0.97(0.81-1.17) | -0.40(-1.71-0.90) | 1.04(0.62-1.74) |
| 35+ | **-2.29(-3.59--0.99)** | 1.13(0.96-1.34) | **-1.38(-2.04--0.71)** | 1.00(0.78-1.29) |
| **ALL WOMEN** |  |  |  |  |
| **No. children (ref:2)** |  |  |  |  |
| 0 | 0.52(-0.71-1.74) | 1.00(0.90-1.1) | -0.40(-0.97-0.17) | 1.05(0.85-1.29) |
| 1 | -0.40(-1.09-0.28) | **1.08(1.02-1.14)** | **-0.68(-1.15--0.20)** | 1.06(0.78-1.44) |
| 3 | -0.17(-1.01-0.67) | 1.03(0.99-1.06) | **-0.76(-1.36--0.16)** | 1.05(0.86-1.29) |
| 4+ | -0.39(-1.19-0.41) | 1.02(0.96-1.09) | **-0.97(-1.82--0.13)** | 1.25(0.96-1.63) |
| **MOTHERS** |  |  |  |  |
| **No. children (ref:2)** |  |  |  |  |
| 1 | -0.59(-1.31-0.13) | **1.07(1.01-1.13)** | **-0.68(-1.14--0.22)** | 1.06(0.76-1.47) |
| 3 | -0.15(-1.02-0.73) | 1.03(0.99-1.07) | **-0.74(-1.36--0.12)** | 1.06(0.86-1.31) |
| 4+ | -0.34(-1.22-0.54) | 1.03(0.97-1.10) | **-0.87(-1.71--0.03)** | 1.27(0.95-1.70) |
| **AFB (ref:** 20-29 years) |  |  |  |  |
| <20 years | 0.06(-0.68-0.81) | 0.99(0.94-1.04) | **-0.55(-0.95--0.15)** | 0.95(0.73-1.24) |
| 30+ | **0.63(0.08-1.18)** | 1.02(0.96-1.08) | -0.08(-0.41-0.24) | 1.05(0.93-1.18) |

Adjusted for: age (continuous), age squared, country fixed-effects, father’s occupation, education, marital status, parity (parent’s models), smoking behaviour, physical activity, household wealth. Depression additionally adjusted for physical health (functional limitations). Coefficients in bold are statistically significant at the 5% level.

**Figures S1a, S1b: Adjusted cross-sectional associations between fertility history and chronic diseases at baseline among men and women aged 50-79 years, SHARE wave 1 (complete case, weighted)**

**
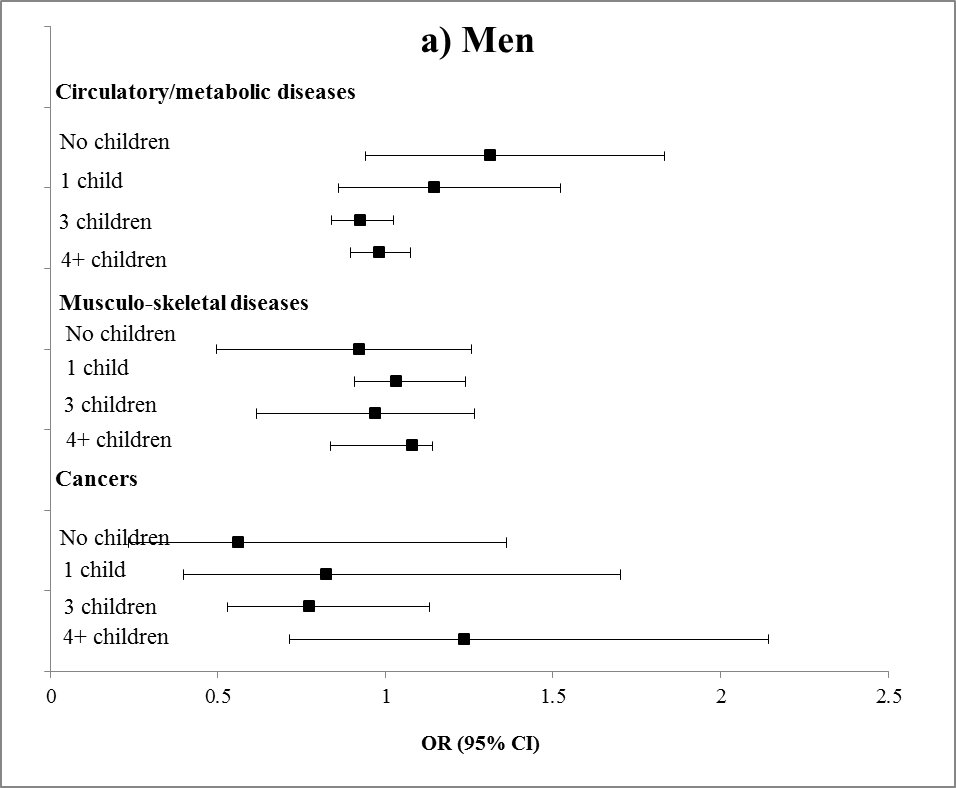

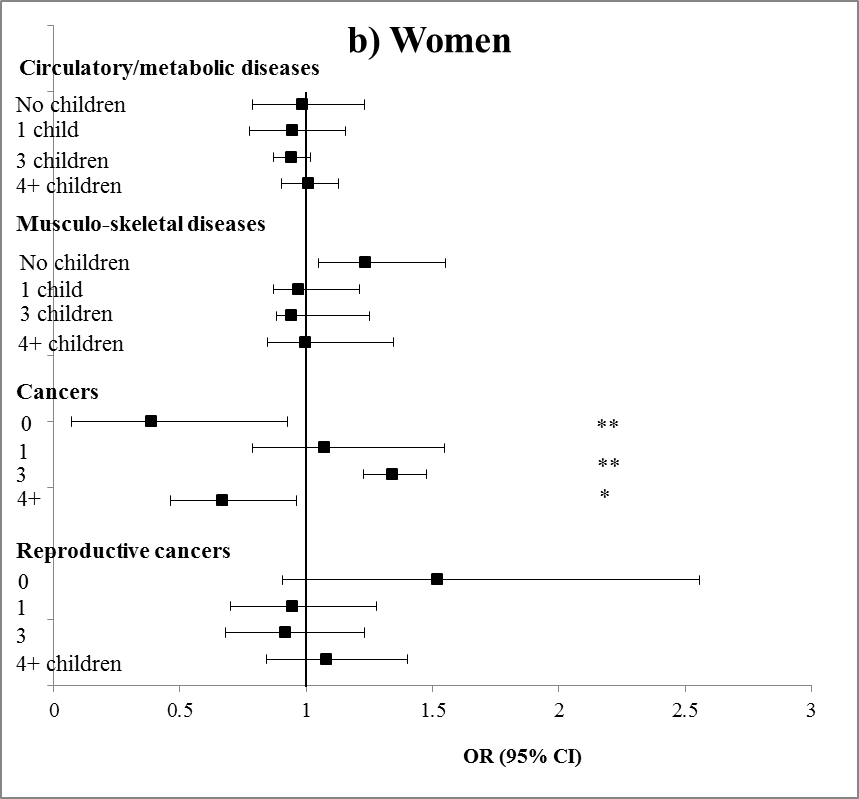
**

***P<0.05 **P<0.01 ***P<0.001**

**Figure S2a and S2b: Adjusted cross-sectional associations between fertility history and chronic diseases at baseline among parents aged 50-79 years, SHARE wave 1 (complete case, weighted)**

**
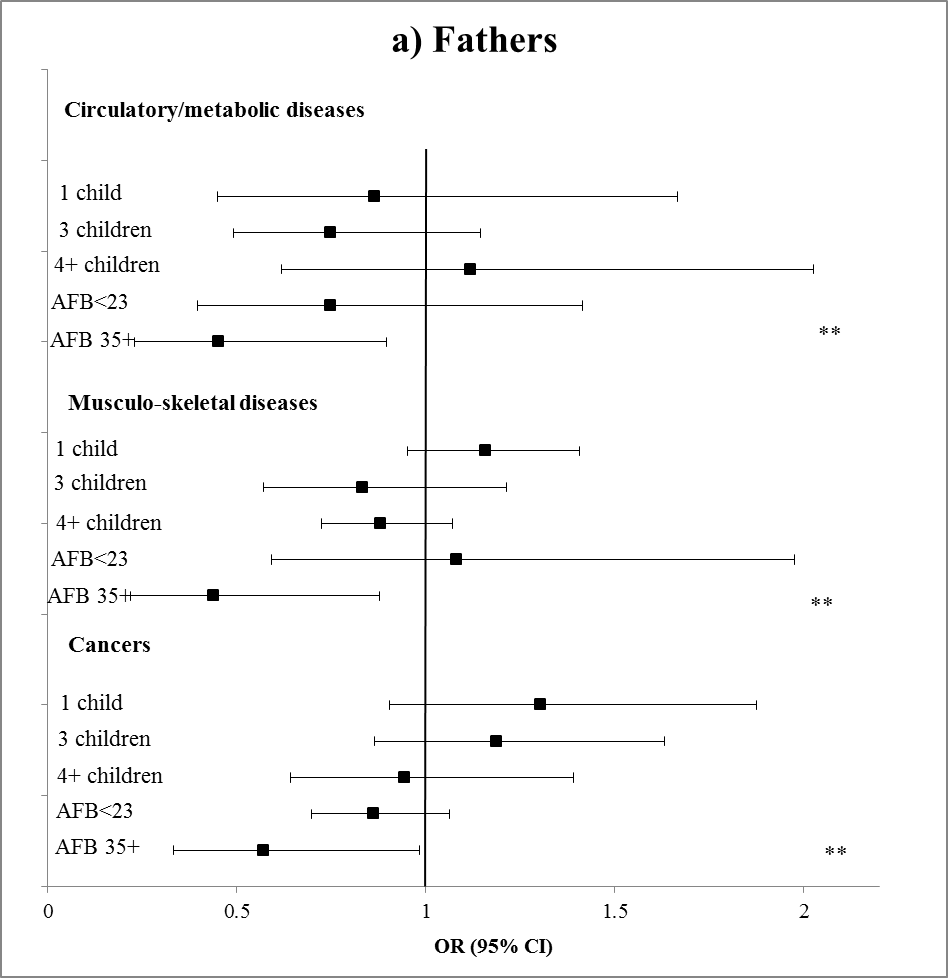

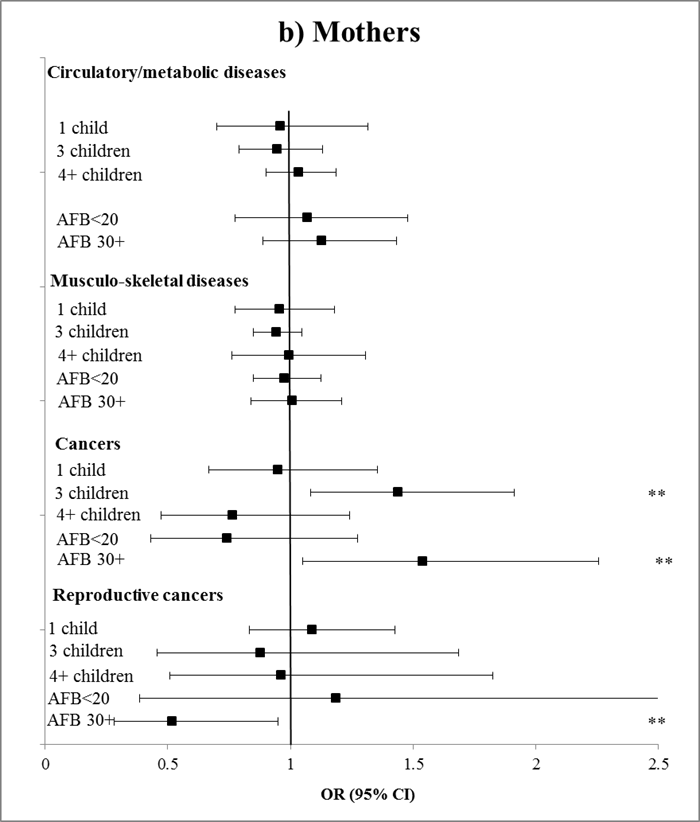
**

***P<0.05 **P<0.01 ***P<0.001**

**Table S3: Adjusted longitudinal associations between fertility history and health outcomes at follow-up (wave 2) among men aged 50-79 years, SHARE wave 1-2**

| ALL MEN | **Grip strength**  **β(95% CI)** | **Functional limitations**  **IRR (95% CI)** | **Depression**  **IRR (95% CI)** | **Cognition**  **β( (95% CI)** | **Index of health conditions**  **OR (95% CI)** |
| --- | --- | --- | --- | --- | --- |
| No. children (ref:2) |  |  |  |  |  |
| 0 | -0.39(-1.61-0.83) | 1.03(0.79-1.33) | 1.29(0.99-1.68) | 0.12(-1.25-1.50) | 0.98(0.82-1.17) |
| 1 | -0.35(-1.01-0.31) | 1.10(0.93-1.03) | 1.13(0.92-1.39) | -0.13(-1.04-0.78) | 1.33(0.95-1.86) |
| 3 | -0.49(-1.51-0.54) | 1.06(0.96-1.18) | 1.12(0.98-1.26) | -0.35(-1.86-1.17) | 1.21(0.92-1.58) |
| 4+ | -0.79(-1.84-0.26) | 1.02(0.87-1.18) | **1.10(1.00-1.22)** | -0.32(-1.68-1.04) | 1.20(0.95-1.51) |
| FATHERS |  |  |  |  |  |
| No. children (ref:2) |  |  |  |  |  |
| 1 | -0.34(-1.04-0.37) | 1.10(0.93-1.30) | 1.14(0.94-1.38) | -0.11(-1.05-0.83) | 1.33(0.96-1.86) |
| 3 | -0.50(-1.58-0.57) | 1.03(0.92-1.15) | 1.10(0.98-1.23) | -0.35(-1.94-1.24) | 1.19(0.91-1.56) |
| 4+ | -0.78(-1.97-0.41) | 0.97(0.84-1.13) | **1.09(1.00-1.19)** | -0.32(-1.72-1.08) | 1.16(0.93-1.44) |
| AFB (ref: 23-34 yrs) |  |  |  |  |  |
| <23 years | -0.54(-1.70-0.62) | **1.27(1.05-1.55)** | 1.04(0.90-1.21) | **-0.88(-1.84-0.08)** | 1.24(0.94-1.63) |
| 35+ | -0.21(-0.79-0.38) | 0.90(0.74-1.09) | 0.94(0.86-1.03) | 0.08(-0.80-0.95) | 0.92(0.73-1.16) |
| ALL WOMEN |  |  |  |  |  |
| No. children (ref:2) |  |  |  |  |  |
| 0 | **0.51(-0.01-1.04)** | 1.01(0.88-1.17) | 1.04(0.99-1.10) | 0.35(-0.38-1.08) | 0.93(0.69-1.26) |
| 1 | **0.40(-0.01-0.800)** | 1.04(0.91-1.20) | 1.03(0.97-1.09) | -0.08(-0.52-0.36) | 1.02(0.88-1.18) |
| 3 | -0.05(-0.68-0.57) | 0.96(0.86-1.08) | 1.00(0.93-1.08) | -0.59(-1.06--0.12) | 1.08(0.91-1.29) |
| 4+ | -0.17(-0.72-0.37) | 1.06(0.94-1.19) | 1.04(0.97-1.11) | **-0.85(-1.37--0.34)** | **1.20(1.07-1.34)** |
| MOTHERS |  |  |  |  |  |
| No. children (ref:2) |  |  |  |  |  |
| 1 | **0.36(0.03-0.70)** | 1.04(0.93-1.17) | 1.02(0.97-1.08) | -0.13(-0.65-0.40) | 1.04(0.93-1.16) |
| 3 | -0.02(-0.74-0.69) | 0.96(0.85-1.07) | 1.00(0.93-1.08) | **-0.61(-1.03--0.19)** | 1.07(0.90-1.27) |
| 4+ | -0.17(-0.67-0.33) | 1.03(0.92-1.15) | 1.03(0.96-1.10) | **-0.84(-1.35--0.32)** | **1.15(1.02-1.31)** |
| AFB (ref: 20-29 yrs) |  |  |  |  |  |
| <20 years | 0.25(-0.08-0.59) | **1.15(1.08-1.23)** | 1.08(0.98-1.18) | -0.36(-1.02-0.300) | **1.27(1.13-1.43)** |
| 30+ | 0.18(-0.11-0.46) | 1.01(0.91-1.14) | 1.03(0.99-1.07) | 0.07(-0.77-0.90) | 0.97(0.84-1.11) |

Adjusted for: health at baseline, months between wave 1 and wave 2, age (continuous), age squared, country fixed-effects, father’s occupation, education, marital status, parity (parent’s models), smoking behaviour, physical activity, household wealth. Depression additionally adjusted for physical health (functional limitations). Bold indicates statistically significant at the 5% level.

**Figure S3a and S3b: Adjusted longitudinal associations between fertility history and chronic diseases in men and women aged 50-79 years, SHARE wave 1-2(complete case, weighted)**


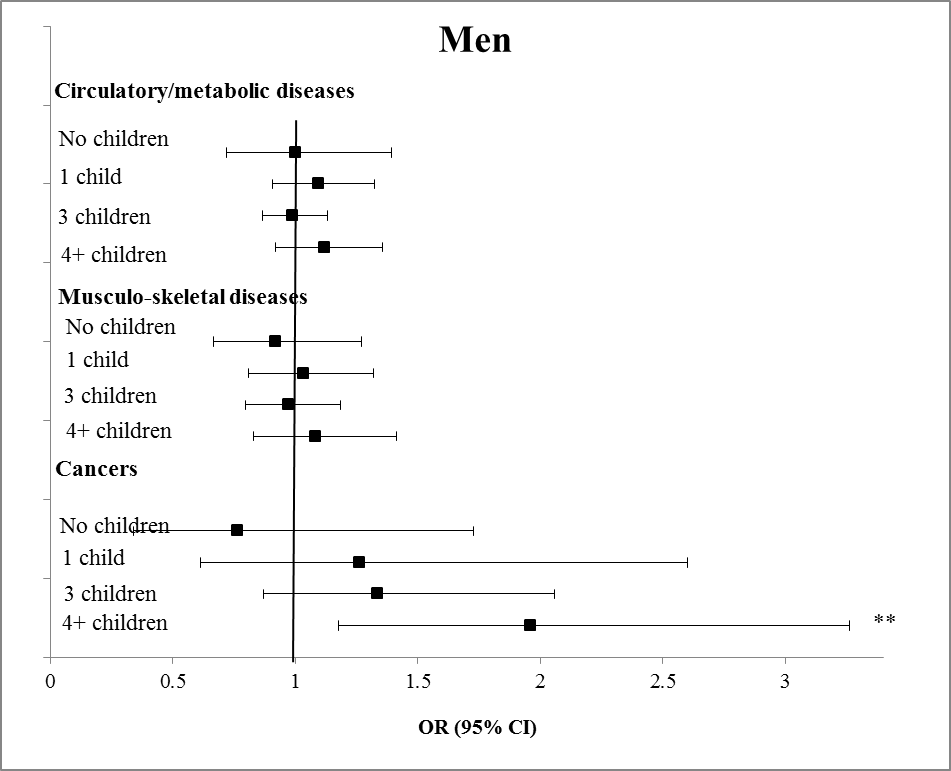

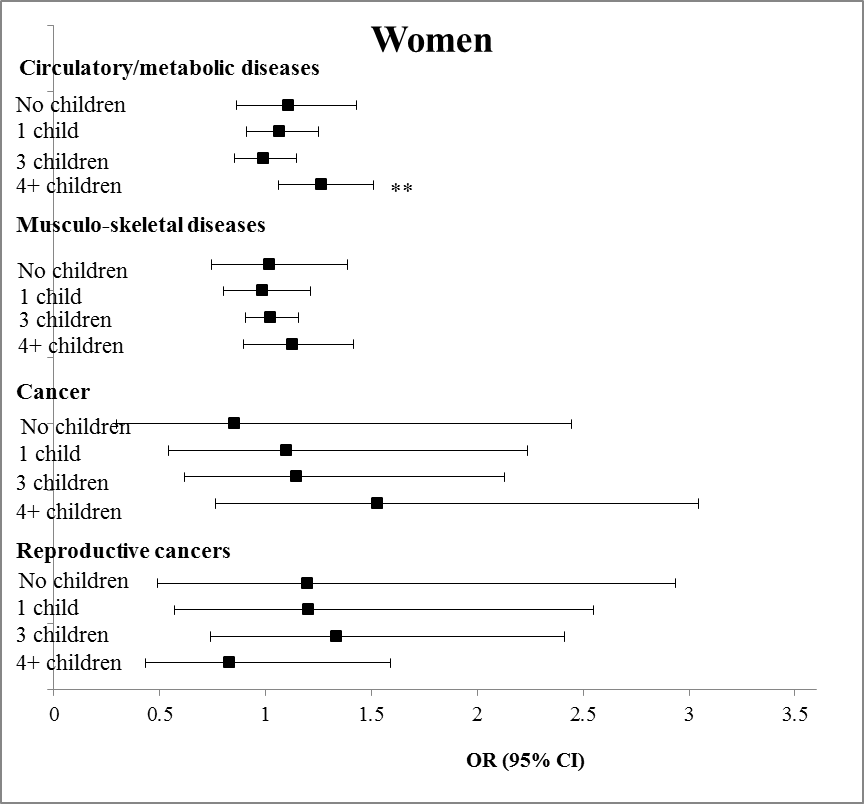
***P<0.05 **P<0.01 ***P<0.001**

**Figure S4a and S4b: Adjusted longitudinal associations between fertility history and chronic diseases in parents aged 50-79 years, SHARE wave 1-2, (complete case, weighted)**


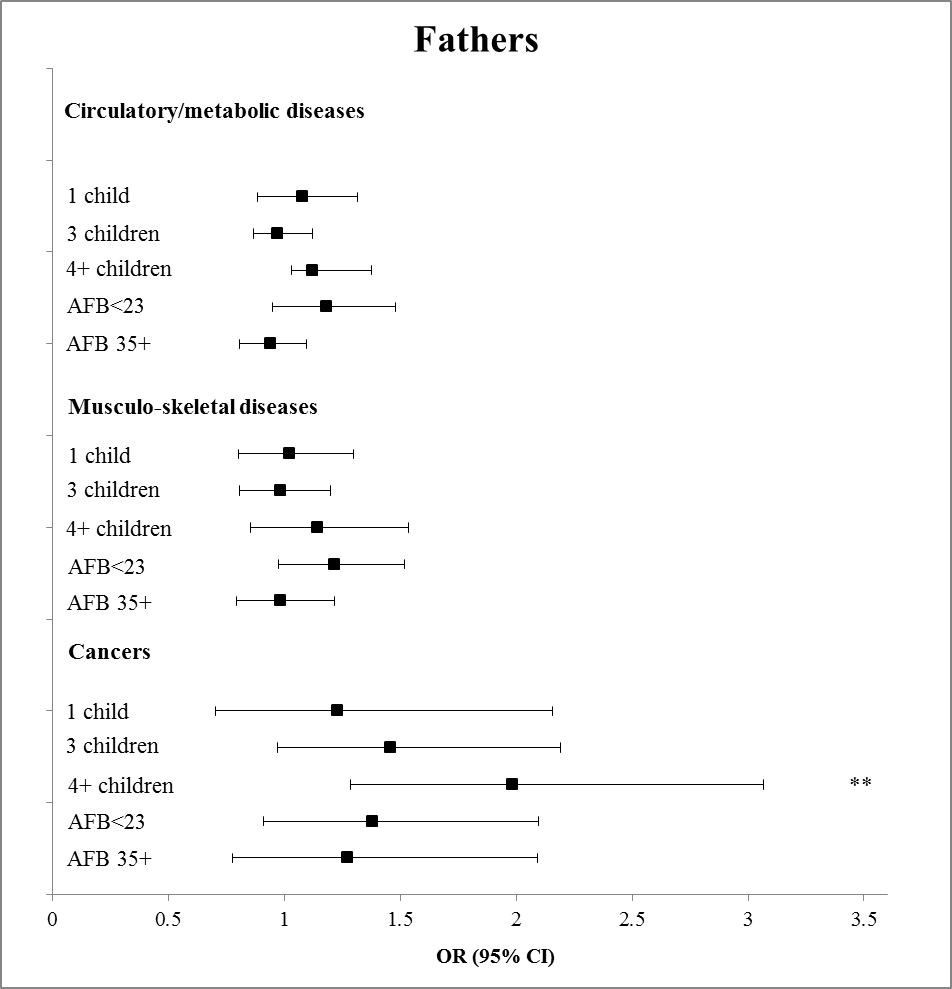

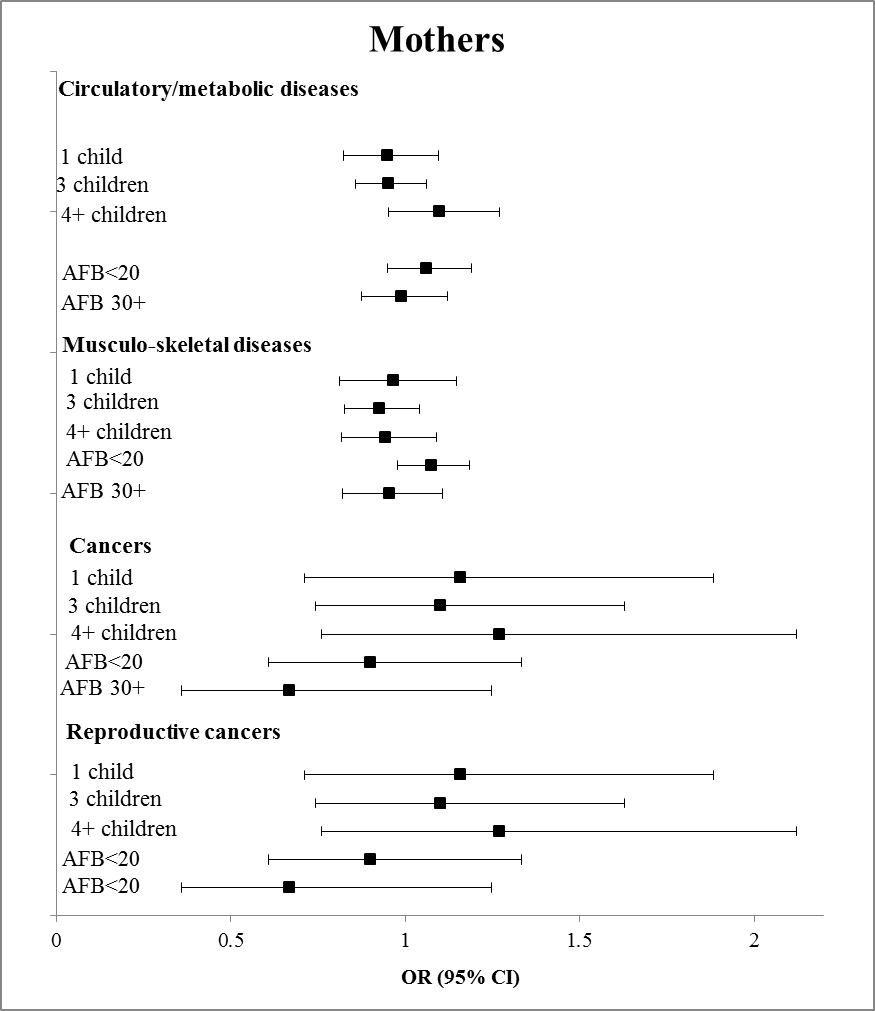
.***P<0.05 **P<0.01 ***P<0.001**

**Figure S5a and S5b: Predicted health index score at wave 1 according to parity and societal/welfare regime in men and women aged 50-79 years, SHARE wave 1 (complete case, weighted)**

**Figure S6a and S6b: Predicted health index score according to parity and marital status in men aged 50-79 years, SHARE wave 1-2 (complete case, weighted)**

**Table S4: Sensitivity analysis adjusting for deceased children: Adjusted cross-sectional associations between fertility history and health outcomes at baseline among men and women aged 50-79 years, SHARE wave 1-3 (complete case analysis, unweighted)**

| **ALL MEN** | **Grip strength**  **β(95% CI)** | **Functional limitations**  **IRR (95% CI)** | **Depression**  **IRR (95% CI)** | **Cognition**  **β( (95% CI)** | **Circulatory and metabolic diseases**  **OR( (95 % CI)** | **Musculoskeletal diseases**  **OR (95% CI)** | **Cancers**  **OR( (95% CI)** |
| --- | --- | --- | --- | --- | --- | --- | --- |
| **No. children (ref:2)** |  |  |  |  |  |  |  |
| 0 | -0.85(-2.23-0.52) | 1.04(0.83-1.30) | 1.05(0.95-1.16) | 0.16(-0.87-1.19) | 1.17(0.91-1.52) | 1.24(0.81-1.89) | 0.85(0.48-1.48) |
| 1 | -0.12(-0.59-0.36) | 1.13(0.97-1.32) | 0.98(0.90-1.06) | 0.38(-0.48-1.25) | 0.98(0.82-1.17) | 1.12(0.90-1.40) | 0.85(0.53-1.38) |
| 3 | 0.01(-0.99-1.00) | 1.07(0.95-1.20) | 1.03(0.96-1.12) | 0.08(-0.66-0.82) | **0.87(0.77-0.98)** | 0.90(0.64-1.25) | 0.61(0.27-1.38) |
| 4+ | -0.22(-1.30-0.86) | **1.19(1.05-1.34)** | **1.11(1.03-1.19)** | 0.02(-1.11-1.16) | 0.86(0.71-1.04) | 1.05(0.85-1.29) | 1.03(0.60-1.76) |
| N | 4486 | 4847 | 4794 | 4804 | 4856 | 4856 | 4623 |
| **FATHERS** |  |  |  |  |  |  |  |
| **No. children (ref:2)** |  |  |  |  |  |  |  |
| 1 | 0.07(-0.39-0.52) | 1.11(0.95-1.30) | 0.96(0.88-1.05) | 0.47(-0.38-1.33) | 1.04(0.85-1.27) | 1.20(0.97-1.50) | 0.87(0.55-1.35) |
| 3 | -0.06(-1.06-0.95) | 1.05(0.94-1.19) | 1.03(0.95-1.12) | 0.05(-0.67-0.77) | **0.86(0.76-0.97)** | 0.86(0.60-1.23) | 0.59(0.26-1.36) |
| 4+ | -0.32(-1.34-0.7) | **1.14(1.00-1.31)** | **1.10(1.01-1.20)** | 0.01(-1.17-1.19) | **0.81(0.69-0.95)** | 1.00(0.80-1.25) | 0.95(0.54-1.65) |
| **AFB (ref:** 23-34 yrs) |  |  |  |  |  |  |  |
| <23 years | -0.10(-1.42-1.23) | 1.15(0.97-1.36) | 1.06(0.93-1.20) | -0.18(-1.22-0.85) | 1.16(0.89-1.52) | 0.99(0.60-1.62) | 1.31(0.83-2.06) |
| 35+ | **-1.35(-2.73-0.03)** | **1.10(1.01-1.20)** | **1.12(1.00-1.27)** | **-0.79(-1.38--0.20)** | **0.67(0.59-0.76)** | 0.57(0.31-1.07) | 0.75(0.47-1.18) |
| N | 3999 | 4303 | 4259 | 4266 | 4310 | 4310 | 4310 |
| **ALL WOMEN** |  |  |  |  |  |  |  |
| **No. children (ref:2)** |  |  |  |  |  |  |  |
| 0 | -0.16(-1.08-0.76) | 1.01(0.91-1.12) | 1.04(0.96-1.12) | -0.33(-0.97-0.32) | 0.96(0.70-1.30) | 1.03(0.75-1.40) | 1.16(0.84-1.59) |
| 1 | -0.12(-0.34-0.09) | 1.05(0.93-1.18) | 1.05(1.00-1.10) | -0.36(-1.13-0.40) | 1.02(0.84-1.24) | 0.91(0.77-1.07) | 0.85(0.54-1.32) |
| 3 | 0.33(-0.15-0.81) | 1.05(0.99-1.11) | 1.01(0.95-1.07) | -0.23(-1.02-0.56) | 0.94(0.84-1.05) | 0.92(0.78-1.07) | 1.02(0.82-1.27) |
| 4+ | 0.21(-0.65-1.07) | **1.26(1.09-1.45)** | 1.04(0.96-1.13) | -0.30(-1.19-0.59) | 0.99(0.88-1.12) | 0.98(0.84-1.14) | 0.99(0.69-1.43) |
| N | 5303 | 5885 | 5837 | 5852 | 5895 | 5895 | 5895 |
| **MOTHERS** |  |  |  |  |  |  |  |
| **No. children (ref:2)** |  |  |  |  |  |  |  |
| 1 | -0.27(-0.58-0.03) | 1.07(0.95-1.20) | 1.06(1.00-1.12) | -0.40(-1.16-0.35) | 1.06(0.86-1.32) | 0.94(0.78-1.14) | 0.86(0.54-1.38) |
| 3 | 0.38(-0.13-0.89) | 1.03(0.96-1.09) | 1.01(0.95-1.07) | -0.19(-0.96-0.59) | 0.93(0.83-1.04) | 0.90(0.78-1.03) | 1.03(0.83-1.29) |
| 4+ | 0.38(-0.59-1.34) | **1.20(1.05-1.36)** | 1.03(0.95-1.11) | -0.18(-1.11-0.75) | 0.96(0.82-1.12) | 0.93(0.77-1.12) | 1.02(0.67-1.54) |
| **AFB (ref:** 20-29 yrs) |  |  |  |  |  |  |  |
| <20 years | **-0.57(-1.03--0.12)** | **1.32(1.19-1.48)** | 1.05(0.98-1.14) | -0.45(-1.09-0.20) | 1.29(1.04-1.60) | 1.18(0.95-1.47) | 0.73(0.52-1.01) |
| 30+ | 0.47(-0.18-1.11) | 1.01(0.94-1.08) | 0.97(0.91-1.03) | 0.02(-0.31-0.35) | 1.00(0.84-1.20) | 0.87(0.68-1.10) | 0.83(0.61-1.14) |
| N | 4790 | 5299 | 5262 | 5275 | 5309 | 5309 | 5309 |

Bold indicates statistically significant at the 5% level.

**Table S5: Sensitivity analysis adjusting for deceased children: Adjusted longitudinal associations between fertility history and health outcomes at baseline among men and women aged 50-79 years, SHARE wave 1-3 (complete case analysis, unweighted)**

| **ALL MEN** | **Grip strength**  **β(95% CI)** | **Functional limitations**  **IRR (95% CI)** | **Depression**  **IRR (95% CI)** | **Cognition**  **β( (95% CI)** | **Circulatory and metabolic diseases**  **OR( (95 % CI)** | **Musculoskeletal diseases**  **OR (95% CI)** | **Cancers**  **OR( (95% CI)** |
| --- | --- | --- | --- | --- | --- | --- | --- |
| **No. children (ref:2)** |  |  |  |  |  |  |  |
| 0 | **-0.84(-1.57--0.11)** | 1.13(0.82-1.56) | 1.11(0.97-1.26) | -0.86(-2.19-0.46) | 0.8(0.58-1.10) | 0.98(0.60-1.57) | 0.81(0.37-1.77) |
| 1 | -0.65(-1.59-0.28) | 1.09(1.00-1.18) | 1.07(0.94-1.22) | -0.15(-0.87-0.56) | 1.06(0.97-1.15) | 1.11(0.91-1.34) | 1.12(0.67-1.87) |
| 3 | -0.61(-1.25-0.04) | 1.1(0.99-1.23) | 1.05(0.97-1.12) | -0.64(-1.36-0.09) | 0.99(0.88-1.10) | 1.00(0.80-1.25) | 0.73(0.43-1.24) |
| 4+ | **-1.06(-1.67--0.45)** | **1.17(1.08-1.27)** | **1.11(1.05-1.17)** | -0.63(-1.53-0.27) | 1.06(0.87-1.29) | 1.14(0.96-1.36) | 1.29(0.91-1.82) |
| N | 4486 | 4847 | 4729 | 4738 | 4856 | 4856 | 4856 |
| **FATHERS** |  |  |  |  |  |  |  |
| **No. children (ref:2)** |  |  |  |  |  |  |  |
| 1 | -0.73(-1.65-0.18) | 1.09(0.99-1.20) | 1.07(0.94-1.22) | -0.20(-0.90-0.50) | 1.07(0.98-1.17) | 1.11(0.95-1.31) | 1.06(0.66-1.69) |
| 3 | -0.60(-1.26-0.06) | 1.08(0.99-1.19) | 1.04(0.97-1.12) | -0.62(-1.36-0.13) | 0.98(0.88-1.10) | 0.98(0.79-1.21) | 0.73(0.44-1.21) |
| 4+ | **-1.03(-1.63--0.42)** | **1.13(1.05-1.22)** | **1.11(1.05-1.18)** | -0.61(-1.45-0.23) | 1.04(0.85-1.26) | 1.09(0.92-1.28) | 1.26(0.87-1.83) |
| **AFB (ref:** 23-34 yrs) |  |  |  |  |  |  |  |
| <23 years | 0.14(-0.86-1.15) | **1.22(1.04-1.42)** | 1.01(0.93-1.09) | -0.71(-1.52-0.10) | 1.10(0.86-1.39) | **1.42(1.09-1.85)** | 1.19(0.70-2.05) |
| 35+ | **0.64(0.11-1.16)** | 0.96(0.77-1.18) | **0.93(0.88-0.99)** | 0.25(-0.28-0.78) | 0.91(0.81-1.03) | 0.99(0.82-1.19) | 1.28(0.75-2.17) |
| N | 3999 | 4303 | 4202 | 4212 | 4310 | 4310 | 4310 |
| **ALL WOMEN** |  |  |  |  |  |  |  |
| **No. children (ref:2)** |  |  |  |  |  |  |  |
| 0 | -0.09(-0.98-0.80) | 0.93(0.82-1.06) | 0.97(0.93-1.02) | -0.08(-1.02-0.86) | 0.93(0.75-1.16) | 1.18(0.92-1.53) | 0.89(0.56-1.41) |
| 1 | 0.33(-0.20-0.87) | 0.99(0.91-1.08) | 0.97(0.92-1.01) | -0.04(-0.69-0.62) | 1.05(0.92-1.20) | 1.04(0.85-1.27) | 0.79(0.53-1.17) |
| 3 | 0.02(-0.48-0.52) | **0.93(0.86-1.00)** | 0.95(0.89-1.01) | **-0.49(-0.93--0.05)** | 1.00(0.83-1.20) | 0.99(0.84-1.15) | 0.77(0.55-1.08) |
| 4+ | 0.14(-0.32-0.60) | 0.97(0.87-1.08) | 1.03(0.97-1.08) | -0.47(-1.18-0.24) | 1.15(0.88-1.49) | 1.10(0.85-1.43) | 0.79(0.54-1.17) |
| N | 5303 | 5885 | 5765 | 5789 | 5895 | 5895 | 5895 |
| **MOTHERS** |  |  |  |  |  |  |  |
| **No. children (ref:2)** |  |  |  |  |  |  |  |
| 1 | 0.31(-0.15-0.77) | 0.98(0.91-1.07) | 0.96(0.93-1.00) | -0.04(-0.68-0.61) | 1.05(0.91-1.21) | 1.03(0.83-1.27) | 0.75(0.49-1.14) |
| 3 | 0.00(-0.50-0.51) | **0.93(0.86-1.00)** | 0.95(0.89-1.02) | **-0.47(-0.86--0.07)** | 1.00(0.81-1.23) | 0.97(0.83-1.12) | 0.77(0.55-1.07) |
| 4+ | 0.14(-0.28-0.56) | 0.96(0.86-1.07) | 1.02(0.97-1.08) | -0.41(-1.09-0.28) | 1.14(0.85-1.52) | 1.05(0.81-1.36) | 0.75(0.44-1.29) |
| **AFB (ref:** 20-29 yrs) |  |  |  |  |  |  |  |
| <20 years | 0.22(-0.13-0.56) | **1.12(1.01-1.24)** | 1.06(0.98-1.15) | -0.31(-0.90-0.28) | 1.12(0.90-1.39) | 1.08(0.88-1.32) | 1.16(0.61-2.20) |
| 30+ | 0.19(-0.48-0.85) | 1.06(0.98-1.14) | 1.04(0.99-1.09) | 0.07(-0.49-0.62) | 1.02(0.87-1.21) | 0.94(0.81-1.09) | 1.04(0.65-1.67) |
| N | 4790 | 5299 | 5196 | 5218 | 5309 | 5309 | 5309 |

Bold indicates statistically significant at the 5% level.

**Table S6: Sensitivity analysis using pattern mixture models: Adjusted longitudinal associations between fertility history and grip strength and cognition among men and women aged 50-79 years, SHARE wave 1-2**

| **ALL MEN (N=9805)** | **Grip strength**  **β** | | | **Cognition score**  **β** | | | **Functional limitations**  **IRR** | | | **Depression score**  **IRR** | | |
| --- | --- | --- | --- | --- | --- | --- | --- | --- | --- | --- | --- | --- |
| **No. children (ref:2)** | **MAR** | **-20%** | **-30%** | **MAR** | **-20%** | **-30%** | **MAR** | **-20%** | **-30%** | **MAR** | **-20%** | **-30%** |
| 0 | 0.12 | -0.45 | -0.83 | 0.11 | -0.27 | -0.51 | 1.03 | 1.04 | 1.05 | 1.06 | 1.07 | 1.08 |
| 1 | 0.41 | -0.03 | -0.32 | 0.13 | -0.27 | -0.54 | 0.99 | 0.99 | 1.00 | 1.02 | 1.03 | 1.04 |
| 3 | 0.48 | 0.45 | 0.42 | 0.05 | 0.00 | -0.03 | 0.94 | 0.94 | 0.94 | 0.93 | 0.94 | 0.94 |
| 4+ | -0.12 | 0.11 | 0.25 | -0.15 | 0.07 | 0.22 | 1 | 1 | 0.99 | 0.99 | 0.99 | 0.98 |
| **FATHERS (N=8903)** |  |  |  |  |  |  |  |  |  |  |  |  |
| **No. children (ref:2)** |  |  |  |  |  |  |  |  |  |  |  |  |
| 1 | 0.28 | -0.25 | -0.60 | 0.01 | -0.42 | -0.70 | 1.00 | 1.00 | 1.01 | 1.03 | 1.04 | 1.05 |
| 3 | 0.43 | 0.37 | 0.33 | 0.08 | 0.02 | -0.03 | 0.96 | 0.96 | 0.96 | 0.93 | 0.93 | 0.94 |
| 4+ | -0.24 | 0.06 | 0.25 | -0.24 | 0.04 | 0.22 | 0.98 | 0.97 | 0.97 | 0.98 | 0.98 | 0.98 |
| **AFB (ref:** 23-34 yrs) |  |  |  |  |  |  |  |  |  |  |  |  |
| <23 years | -0.45 | -0.87 | **-1.15*** | -0.30 | -0.65 | **-0.87*** | 1.16 | 1.17 | 1.17 | 1.04 | 1.04 | 1.05 |
| 35+ | 0.12 | 0.36 | 0.52 | -0.21 | -0.05 | 0.06 | 0.94 | 0.94 | 0.94 | 0.94 | 0.94 | 0.94 |
| **ALL WOMEN (N=11439)** |  |  |  |  |  |  |  |  |  |  |  |  |
| **No. children (ref:2)** |  |  |  |  |  |  |  |  |  |  |  |  |
| 0 | 0.20 | -0.26 | -0.55 | 0.61 | 0.27 | 0.05 | 1.03 | 1.04 | 1.05 | 1.03 | 1.04 | 1.04 |
| 1 | 0.29 | -0.03 | -0.24 | 0.29 | -0.08 | -0.33 | 1.07 | 1.08 | 1.08 | 1.02 | 1.03 | 1.04 |
| 3 | 0.18 | 0.00 | -0.12 | 0.39 | 0.18 | 0.05 | 1.06 | 1.07 | 1.07 | 1.00 | 1.00 | 1.01 |
| 4+ | -0.07 | -0.20 | -0.28 | -0.24 | -0.29 | -0.33 | **1.09*** | 1.09 | 1.09 | 1.04 | 1.04 | 1.05 |
| **MOTHERS (N=10,373)** |  |  |  |  |  |  |  |  |  |  |  |  |
| **No. children (ref:2)** |  |  |  |  |  |  |  |  |  |  |  |  |
| 1 | 0.16 | 0.16 | 0.06 | 0.30 | 0.10 | -0.04 | 1.10 | 1.12 | 1.13 | 1.03 | 1.04 | 1.05 |
| 3 | -0.02 | -0.02 | -0.07 | 0.39 | 0.28 | 0.20 | 1.06 | 1.07 | 1.08 | 0.99 | 1.00 | 1.00 |
| 4+ | -0.05 | -0.05 | -0.09 | -0.22 | -0.24 | -0.25 | 1.09 | 1.08 | 1.09 | 1.04 | 1.05 | 1.05 |
| **AFB (ref:** 20-29 yrs) |  |  |  |  |  |  |  |  |  |  |  |  |
| <20 years | 0.02 | 0.02 | -0.05 | 0.01 | -0.09 | -0.16 | 1.12 | 1.12 | 1.13 | 1.06 | 1.06 | 1.07 |
| 30+ | -0.04 | -0.04 | -0.05 | 0.06 | 0.07 | 0.08 | 0.99 | 0.99 | 0.99 | 1.00 | 0.99 | 0.99 |

*P<0.05 **P<0.005. ***P<0.001 Adjusted for: health at baseline, months between wave 1 and wave 2, age (continuous), country fixed-effects, father’s occupation, education, marital status, parity (parent’s models), smoking behaviour, physical activity, household wealth. Depression additionally adjusted for physical health (functional limitations).

**Table S7: Sensitivity analysis using pattern mixture models: Adjusted longitudinal associations between fertility history and chronic diseases among men and women aged 50-79 years, SHARE wave 1-2**

| **ALL MEN (N=9805)** | **Circulatory and metabolic diseases**  **OR** | | | **Musculoskeletal diseases**  **OR** | | | **Cancers**  **OR** | | |
| --- | --- | --- | --- | --- | --- | --- | --- | --- | --- |
| **No. children (ref:2)** | **MAR** | **+20%** | **+30%** | **MAR** | **+20%** | **+30%** | **MAR** | **+20%** | **+30%** |
| 0 | 1.04 | 1.06 | 1.07 | 0.82 | 0.90 | 0.97 | 0.55 | 0.96 | 0.98 |
| 1 | 1.14 | 1.16 | 1.17 | 1.15 | 1.14 | 1.13 | 1.05 | 1.08 | 1.12 |
| 3 | 1.00 | 0.99 | 1.01 | 0.99 | 0.94 | 0.95 | 1.11 | 0.97 | 1.04 |
| 4+ | **1.24*** | **1.18*** | 1.18 | 1.17 | 1.04 | 1.01 | 1.51 | 1.07 | 1.06 |
| **FATHERS (N=8903)** |  |  |  |  |  |  |  |  |  |
| **No. children (ref:2)** |  |  |  |  |  |  |  |  |  |
| 1 | 1.23 | **1.27*** | **1.28*** | 1.15 | 1.19 | 1.25 | 1.08 | 1.23 | 1.30 |
| 3 | 1.01 | 1.04 | 1.02 | 1.01 | 1.00 | 1.00 | 1.36 | 1.12 | 1.06 |
| 4+ | 1.22 | 1.22 | 1.15 | 1.14 | 1.05 | 1.02 | 1.72 | 1.09 | 1.01 |
| **AFB (ref:** 23-34 yrs) |  |  |  |  |  |  |  |  |  |
| <23 years | 1.99 | 1.03 | 1.07 | 1.15 | 1.17 | 1.18 | 1.29 | 1.31 | 1.28 |
| 35+ | 0.96 | 0.97 | 0.94 | 0.97 | 0.96 | 0.93 | 1.79 | 1.19 | 1.05 |
| **ALL WOMEN (N=11439)** |  |  |  |  |  |  |  |  |  |
| **No. children (ref:2)** |  |  |  |  |  |  |  |  |  |
| 0 | 1.01 | 1.06 | 1.06 | 0.89 | 0.97 | 0.96 | 0.90 | 1.24 | 1.19 |
| 1 | 1.03 | 1.06 | 1.05 | 0.98 | 1.02 | 1.01 | 1.87 | 1.33 | 1.28 |
| 3 | 1.02 | 1.04 | 1.02 | 0.96 | 1.01 | 1 | 1.57 | 1.18 | 1.10 |
| 4+ | **1.21*** | **1.21*** | **1.15*** | 1.08 | 1.09 | 1.07 | **2.31**** | 1.14 | 1.09 |
| **MOTHERS (N=10,373)** |  |  |  |  |  |  |  |  |  |
| **No. children (ref:2)** |  |  |  |  |  |  |  |  |  |
| 1 | 1.07 | 1.09 | 1.09 | 1.04 | 1.05 | 1.06 | 1.01 | 1.07 | 1.10 |
| 3 | 1.05 | 1.05 | 1.04 | 1.02 | 1.03 | 1.02 | 1.06 | 1.07 | 1.09 |
| 4+ | **1.26*** | **1.25*** | **1.25*** | 1.03 | 1.02 | 1.02 | 1.39 | 1.17 | 1.21 |
| **AFB (ref:** 20-29 yrs) |  |  |  |  |  |  |  |  |  |
| <20 years | 1.04 | 1.05 | 1.05 | 1.09 | 1.10 | 1.11 | 0.83 | 0.94 | 0.93 |
| 30+ | 1.05 | 1.05 | 1.05 | 0.89 | 0.90 | 0.90 | 1.02 | 0.97 | 0.99 |

*P<0.05 **P<0.005. ***P<0.001 Adjusted for: health at baseline, months between wave 1 and wave 2, age (continuous), country fixed-effects, father’s occupation, education, marital status, parity (parent’s models), smoking behaviour, physical activity, household wealth. Depression additionally adjusted for physical health (functional limitations)
